# Meta-Analysis of Salt Stress Transcriptome Responses in Different Rice Genotypes at the Seedling Stage

**DOI:** 10.3390/plants8030064

**Published:** 2019-03-12

**Authors:** Weilong Kong, Hua Zhong, Ziyun Gong, Xinyi Fang, Tong Sun, Xiaoxiao Deng, Yangsheng Li

**Affiliations:** State Key Laboratory for Hybrid Rice, College of Life Sciences, Wuhan University, Wuhan 430072, China; Weilong.Kong@whu.edu.cn (W.K.); zhonghua0103@whu.edu.cn (H.Z.); Gziyun@whu.edu.cn (Z.G.); 15764210582@163.com (X.F.); stong_fx@whu.edu.cn (T.S.); 2017102040003@whu.edu.cn (X.D.)

**Keywords:** *Oryza sativa* L., salt stress, abiotic stresses, transcriptome responses, MapMan analysis, transcription factors, qRT-PCR

## Abstract

Rice (*Oryza sativa* L.) is one of the most important staple food crops worldwide, while its growth and productivity are threatened by various abiotic stresses, especially salt stress. Unraveling how rice adapts to salt stress at the transcription level is vital. It can provide valuable information on enhancing the salt stress tolerance performance of rice via genetic engineering technologies. Here, we conducted a meta-analysis of different rice genotypes at the seedling stage based on 96 public microarray datasets, aiming to identify the key salt-responsive genes and understand the molecular response mechanism of rice under salt stress. In total, 5559 genes were identified to be differentially expressed genes (DEGs) under salt stress, and 3210 DEGs were identified during the recovery process. The Gene Ontology (GO) enrichment results revealed that the salt-response mechanisms of shoots and roots were different. A close-knit signaling network, consisting of the Ca^2+^ signal transduction pathway, the mitogen-activated protein kinase (MAPK) cascade, multiple hormone signals, transcription factors (TFs), transcriptional regulators (TRs), protein kinases (PKs), and other crucial functional proteins, plays an essential role in rice salt stress response. In this study, many unreported salt-responsive genes were found. Besides this, MapMan results suggested that TNG67 can shift to the fermentation pathway to produce energy under salt stress and may enhance the Calvin cycle to repair a damaged photosystem during the recovery stage. Taken together, these findings provide novel insights into the salt stress molecular response and introduce numerous candidate genes for rice salt stress tolerance breeding.

## 1. Introduction

Since plants are sessile, they are forced to continuously face a multitude of biotic and abiotic stresses during their lifespan. Abiotic stresses, such as salt, drought, and low temperature, seriously threaten the growth and agricultural productivity of plants [1]. Of the abiotic stresses, salt stress is a serious threat to crops’ yield worldwide. According to the FAO Land and Plant Nutrition Management Service, at least 6% of the world’s land is affected by salt stress to different degrees [2]. In recent years, environmental pollution and climate change have intensified the adverse effects of salt stress through raising soil salinity [3]. Previous investigations have clarified that salt stress impairs plants in the form of osmotic stress and oxidative stress [4]. Osmotic stress breaks the selective permeability of the cell membrane and ion homeostasis due to a high-salinity external condition and an excessive accumulation of Na^+^ and Cl^−^ in plant cells, which seriously impede the absorption of water and nutrients in plants [2,5]. Besides this, the imbalance in production and elimination of reactive oxygen species (ROS) leads to subsequent oxidative stress [6,7].

Over the past few decades, many studies have focused on the molecular process of plant salt tolerance based on emerging new technologies, including RNA-sequencing (RNA-seq), alternative splicing analysis, miRNA analysis, epigenetics, and quantitative trait locus (QTL) mapping [8,9,10,11,12,13,14,15]. These studies have demonstrated that various signaling pathways play vital roles during the plant salt stress response process, including the Ca^2+^-mediated signaling pathway, the mitogen-activated protein kinase (MAPK) cascade, the ROS signaling pathway, and the abscisic acid (ABA) signaling pathway [16,17]. In addition, functional protein genes and transcription factors (TFs) were found to be involved in plant salt stress response and tolerance [17].

Rice (*Oryza sativa* L.) is one of the most important staple food crops worldwide and also a model for plant genomic studies in monocots [18]. So far, several studies have reported lots of salt-responsive genes in rice based on microarray or RNA–seq analysis [8,19,20,21,22,23]. For example, transcript changes at the initial phase of salt stress were investigated using a 1728-cDNA library of roots from the salt-tolerant rice (var. Pokkali) [19]. Another study with a cDNA microarray library from shoots containing 9000 unigenes identified 486 salt-responsive expressed sequence tags in the highly salt-tolerant *indica* rice, Nona Bokra [20]. In addition, another study reported that a total of 1676, 817, and 1310 upregulated genes and 1270, 1323, and 2284 downregulated genes were identified in the flag leaf, shoot, and panicle of Minghui 63 (*indica*) under a high-salinity condition, respectively [21]. In 2010, 995 and 1052 genes were identified to be linked to salt stress in Nipponbare (*japonica*) based on RNA-seq, separately [22]. Zhou et al. (2016) conducted an RNA-seq analysis of Dongxiang wild rice (*Oryza rufipogon* Griff), and their study reported 6867 differentially expressed transcripts (2216 upregulated and 4651 downregulated) in the leaves and 4988 differentially expressed transcripts (3105 upregulated and 1883 downregulated) in the roots [23]. Wang et al. (2018) reported that a total of 5273 differentially expressed genes (DEGs) were identified between salt-tolerant and sensitive genotypes of *indica* rice at the seedling stage [8]. In fact, mechanisms of gene regulation are different at different development stages, in various tissues, and in different genotypes [8,24]. Although multiple previous studies have tried to explain the rice regulatory mechanisms of salt tolerance based on microarray or RNA-seq analysis, the potential regulatory mechanism of salt tolerance is still not fully understood, especially the differences in the salt stress tolerance of different genotypes. Thus, the roots’ and shoots’ microarray datasets (GSE76613) of the TNG67 genotype (rice subspecies *indica*, salt-tolerant) and the TCN1 genotype (rice subspecies *japonica*, salt-sensitive) were downloaded and analyzed. This study not only contributes to a better understanding of the molecular mechanisms of salt stress tolerance but also provides candidate genes for salt-resistance molecular breeding.

## 2. Materials and Methods

### 2.1. Plant Materials

The ‘Nipponbare’ rice (*O. sativa* ssp. *japonica*) was chosen for the quantitative real-time RT-PCR (qRT-PCR) verification of randomly selected DEGs. After 2 days of germination in water at 37 ℃, seeds were grown in containers with sponges as supporting materials in Yoshida solution with 60% relative humidity and with a light and temperature regime of 14 h/10 h, light/dark, 30 ℃/22 ℃. Three-leaf stage seedlings were transferred to 200 mM NaCl Yoshida solution for salt treatment. Then, the roots of treatment/control seedlings were collected at 0, 3, 6, 12, and 24 h for RNA extraction. In this study, 0 h was the control group, and 3 h, 6 h, 12 h, and 24 h were treatment groups. Three biological replicates were adopted for each group. For each biological replicate, 15 seedlings were collected and mixed. Totally, 15 RNAs (three control groups and 12 treatment groups) were extracted using the TRIzol method and all RNAs were reverse-transcribed into cDNAs using the PrimeScript RT reagent Kit (TakaRa, Dalian, China).

### 2.2. Data Collection and Meta-Analysis

The series matrix file of the TNG67 genotype (rice subspecies *indica*, salt-tolerant, 48 datasets of the roots and shoots) and the TCN1 genotype (rice subspecies *japonica*, salt-sensitive, 48 datasets of the roots and shoots) were obtained from the Gene Expression Omnibus (GEO) repository (accession number GSE76613; https://www.ncbi.nlm.nih.gov/geo/query/acc.cgi?acc=GSE76613). Within the two genotypes, 0-h, 3-h, 24-h, and recovery 24-h roots and shoots from three-leaf stage seedlings after 250 mM NaCl treatment were used in this study. Detailed information (experiment design, transcriptome analysis, array information, data processing, and platform ID) of GSE76613 can be obtained from the GEO repository, and this information is partly summarized in Table S1. Raw data analysis and ID conversion were performed using the R language package [25,26]. A difference analysis was carried out with the limma package in the Bioconductor package (http://www.bioconductor.org/) [26]. DEGs were considered those with a |log_2_fold change| >1.5 after normalization to the control and with significant results for the t-test (p value <0.05) based on six replicates (3 biological repeats x 2 technical repeats) for each treatment compared with the control [18].

### 2.3. Gene Ontology (GO) Enrichment Analysis

To understand DEGs’ functions, a GO enrichment analysis of core salt-responsive DEGs (common DEGs at 3 h and 24 h of both genotypes in shoots or roots) was implemented by the GOseq R package (https://www.bioconductor.org/packages/release/bioc/html/goseq.html) based on the Wallenius noncentral hyper-geometric distribution [27]. GO terms with a corrected p value of less than 0.001 were considered significantly enriched.

### 2.4. TF, TR, and PK Identification and MapMan Analysis

For the identification of TFs, the DEG sequences were searched against the Plant transcription factor database (PlantTFDB 4.0, http://planttfdb.cbi.pku.edu.cn/) with an E-value cut off of ≤10^−5^ [28]. For the identification of transcriptional regulators (TRs) and protein kinases (PKs), all of the core salt-responsive sequences in roots and shoots were analyzed by iTAK software (http://itak.feilab.net/cgi-bin/itak/index.cgi) [29]. The average log_2_fold change values of 3 biological repeats x 2 technical repeats for each treatment compared with the control at different points were displayed using MapMan 3.6.0 (https://mapman.gabipd.org/) [18,30].

### 2.5. Sequence Alignment and Gene Comparison

Genes that have previously been identified as salt-responsive genes were obtained based on previous papers [8,23,31]. Gene ID conversion between different genome versions (such as 9311 and Nipponbare) was conducted by diamond software (https://ab.inf.uni-tuebingen.de/software/) using the blastp method with the following parameters: max-target-seqs 1, evalue 1e-10 [32]. Common DEGs are shown by a Venn diagram.

### 2.6. DEG Mapping on the Previously Identified Salt-Stress-Related QTL Intervals

A total of 17 salt-stress-related QTLs of rice were downloaded from the Gramene QTL database (http://archive.gramene.org/db/) [23]. All DEGs were mapped on these QTLs according to sequence and QTL location information.

### 2.7. Quantitative Real-Time PCR (qRT-PCR) Validation of DEGs

In this study, six DEGs were randomly selected for the verification of the DEG results. Primers of these genes were designed by Primer 5.0 in specific regions or 3’–UTR regions (the primers in Table S2). The qRT-PCR reaction (10 μL) was formulated using ChamQ™ SYBR^®^ Color qPCR Master Mix (Vazyme, Shanghai, China). qRT-PCR was carried out in 96-well plates on a CFX96 Touch™ Real-Time PCR Detection System (Bio-Rad, Hercules, CA, USA). *Ubi* (*LOC_Os03g13170*, encodes ubiquitin fusion protein) was used as an internal control. The average threshold cycle (Ct) from three biological replicates was used to determine the fold change of gene expression by the 2^−ΔΔCT^ method [33].

## 3. Result

### 3.1. Identification of DEGs Involved in Salt Stress

**Figure 1:**
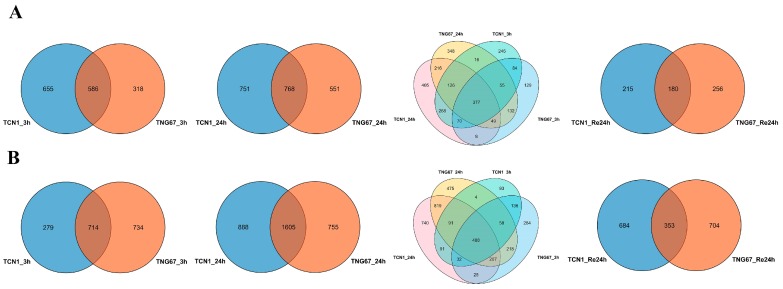
A Venn diagram of differentially expressed genes (DEGs) in shoots of TNG67 and TCN1 seedlings at different points (exposed to salt stress for 3 h or 24 h and allowed to recover for 24 h) (**A**); a Venn diagram of DEGs in roots of TNG67 and TCN1 seedlings at different points (**B**).

Comparisons of gene expression in the shoots from the two genotypes (TNG67 and TCN1) (Table S3) revealed a total of 1241 DEGs in TCN1 and 904 DEGs in TGN67 at 3 h of salt treatment (S3) and two genotypes shared 586 DEGs (Figure 1A). Following 24 h after salt stress (S24), 1519 and 1319 DEGs were identified in TCN1 and TGN67, respectively, and 738 DEGs were shared by two genotypes (Figure 1A). At S3 and S24, two genotypes shared 377 DEGs (Figure 1A). These genes showed similar expression patterns at S3 and S24 in these two genotypes (Figures S2 and S3, Tables S4 and S5), suggesting that these genes were core salt-responsive genes in shoots between the two genotypes. After 24 h recovery after salt stress (ReS24), 395 DEGs were identified in TCN1, while 436 DEGs were identified in TGN67, and the two genotypes had 180 DEGs in common (Figure 1A). In roots, 993 and 1448 DEGs were identified at S3 (714 DEGs in common), while 2493 and 2360 DEGs were identified at S24 (1605 DEGs in common) (Figure 1B). At S3 and S24, a total of 488 DEGs were shared by the two genotypes (Figure 1B). These genes displayed similar expression patterns at S3 and S4 in both genotypes (Figures S2 and S4, Tables S4 and S6), indicating that these genes were core salt-responsive genes in the roots of these two genotypes. Interestingly, a total of 123 DEGs were found between core salt-responsive genes in shoots and core salt-responsive genes in roots (Figure S1 and Table S4). As these genes also showed similar expression patterns in the shoots and roots of the two genotypes at S3 and S24, these genes play important roles during the salt stress process. At ReS24, in roots, 1037 DGEs were identified in TCN1 as well as 1057 DEGs in TGN67, and the two genotypes shared 353 DEGs (Figure 1B). Under salt stress, the number of DEGs identified in roots was greater than that in shoots within these two genotypes (Figure 1), suggesting that the salt stress has a more broad effect on gene expression in roots than in shoots. We also observed that the number of DEGs at S24 was greater than that at S3 in shoots and roots in both genotypes.

**Figure 2:**
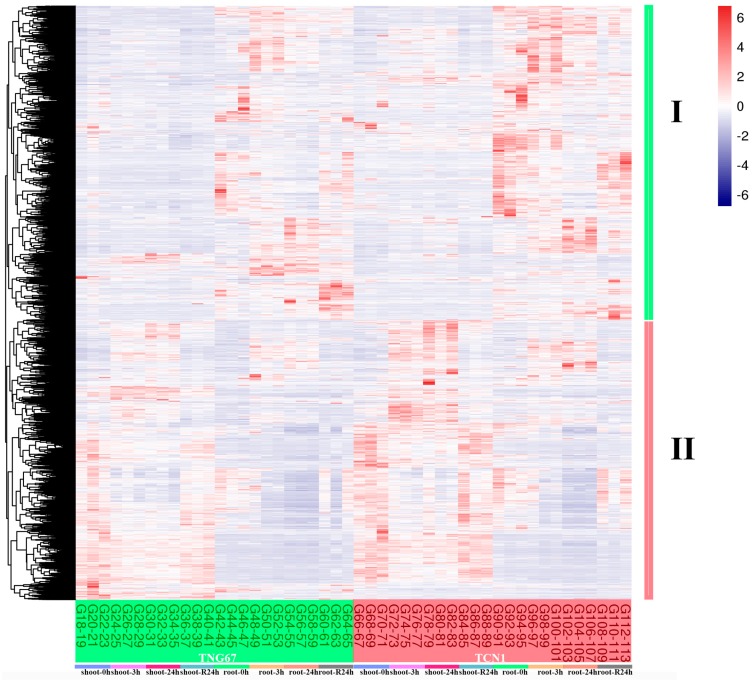
The heat map analysis of all DEGs in shoots and roots of TNG67 and TCN1 at 3 h and 24 h after salt stress treatments and at a subsequent 24-h recovery point.

The heat map of all DEGs revealed that all DEGs showed similar expression patterns in these two genotypes (Figure 2), indicating that the main response mechanism of salt stress was the same in both genotypes. In addition, all DEGs can be grouped into two groups (I and II). Genes in Group I showed higher expression levels in roots than in shoots, while genes in Group II had higher expression levels in shoots than in roots (Figure 2). The core salt-responsive genes in shoots and roots also showed different expression patterns (Figures S3 and S4). These results suggest that the salt-response mechanisms were different between shoots and roots. Thus, the core salt-responsive genes in shoots and roots were annotated by a GO enrichment analysis, respectively.

To verify the reliability of DEGs in this study, six salt-responsive DEGs with significantly upregulated expression levels under salt stress were randomly chosen for qRT-PCR (Figure S5). The qRT-PCR result revealed that these six genes were significantly upregulated after salt stress.

### 3.2. GO Enrichment Analysis of Core Salt-Responsive Genes

To understand the core salt-responsive genes’ functions in shoots or roots, a GO enrichment analysis of these genes was performed. A total of 30 and 25 terms were enriched in shoots and roots, respectively (Table 1 and Table 2). Shoots and roots shared seven terms, including ‘response to cadmium ion (GO:0046686)’, ‘response to water deprivation (GO:0009414)’, ‘response to cold (GO:0009409)’, ‘response to salt stress (GO:0009651)’, ‘hyperosmotic salinity response (GO:0042538)’, ‘response to abscisic acid (GO:0009737)’, and ‘response to wounding (GO:0009611)’. These terms are all related to abiotic stress, especially salt stress. As shown in Table 1 and Table 2, the remaining terms involve multiple biological processes. These results revealed that the salt-response mechanisms were different in shoots and roots. Shoots involve an abscisic acid response, the hydrogen peroxide catabolic process, the glucose catabolic process, the ethylene biosynthetic process, and the others, while roots mainly involve nitrate transport, lipid transport, selenate transport, the salicylic-acid-mediated signaling pathway, and the others.

### 3.3. MapMan Analysis of All Core Salt-Reponsive Genes of Shoots and Roots

**Figure 3:**
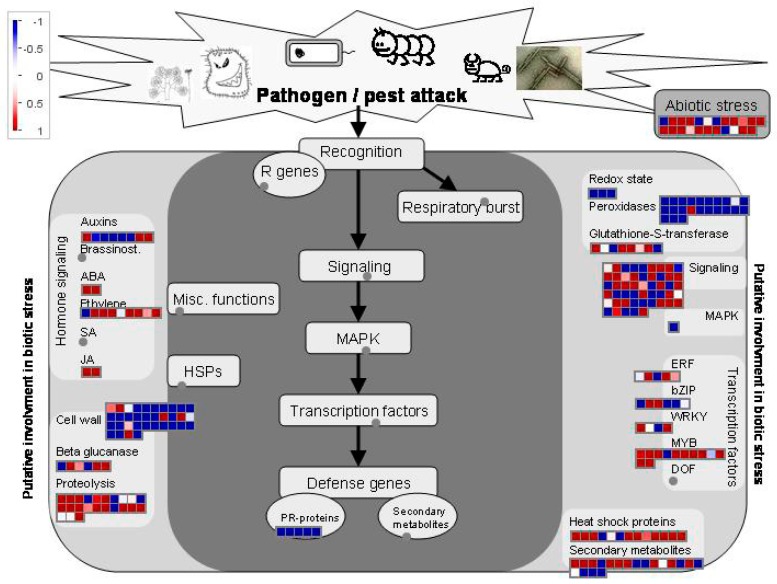
The MapMan biotic stress overview of core salt-responsive genes in shoots after 3 h of salt stress treatment. In addition, 24 h and recovery 24 h in shoots, as well as 3 h, 24 h, and recovery 24 h in roots, are shown in Figure S6. ABA, abscisic acid; MAPK, mitogen-activated protein kinase; SA, salicylic acid; JA, jasmonic acid; HSPs, heat shock proteins.

For a better understanding of core salt-stress-response mechanisms, all core salt-responsive genes of shoots and roots (742 genes) were visualized by a biotic stress overview in MapMan 3.6.0. This result showed that the salt-stress-response mechanisms in rice were very complicated and involved multiple signal transductions, MAPK, TFs, and defense genes (Figure 3). For example, lots of signaling receptor kinase genes, signaling calcium genes, signaling phosphoinositide genes, and signaling G-protein genes were significantly upregulated. Similarly, Auxins, ABA, Ethylene, and jasmonic acid (JA) signaling genes also showed significant upregulations. In addition, we found that ERF-, bZIP-, WRKY-, and MYB-TFs were significantly upregulated. Defense genes, including heat shock protein genes, secondary metabolite genes, and the others, also showed upregulation.

### 3.4. Identification of TFs, TRs, and PKs Involved in Salt Stress

**Figure 4:**
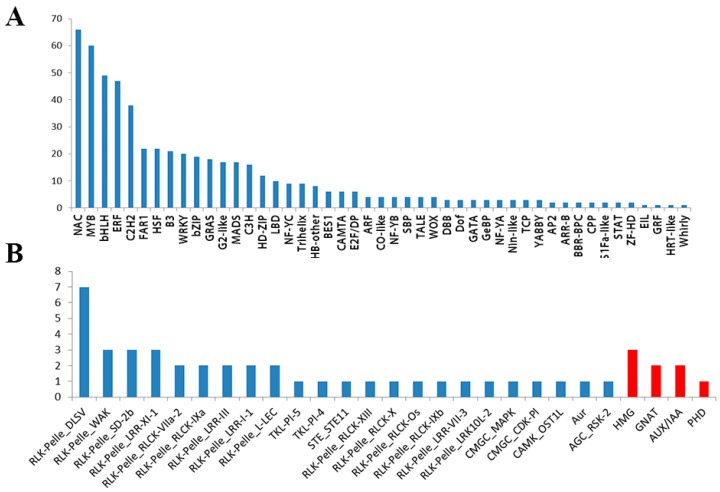
The transcription factor (TF) classification of all core salt-responsive DEGs based on PlantTFDB 4.0 with an E-value cut off of ≤10^−5^ (**A**). The transcriptional regulator (TR) and protein kinase (PK) classifications of all core salt-responsive DEGs were performed by iTAK software (**B**). The Y-axis shows the numbers of TFs, TRs, and PKs.

In order to identify more salt-responsive TFs, TRs, and PKs, we conducted an identification analysis of all core salt-responsive genes of shoots and roots (742 genes) using iTAK software. In this study, a total of 555 DEGs-TFs were identified (74.80%). These contained 47 families, of which the top seven families are NAC (66, 8.9%), MYB (60, 8.1%), bHLH (49, 6.6%), ERF (47, 6.3%), C2H2 (38, 5.1%), FHR1 (22, 3.0%), and HSF (22, 3.0%), respectively (Figure 4A and Table S7). In addition, 23 PK subfamilies and four TR families were identified (Figure 4B). Among them, the majority of PK subfamilies belonged to the receptor-like kinases (RLK/Pelle) family. In TRs, three members of HMG, two members of GNAT, two members of AUX/IAA, and one member of PHD were identified. 

### 3.5. Key Genes Involved in the Response to Salt Stress

#### 3.5.1. DEGs Involved in the Ca^2+^ Signal Transduction Pathway

**Figure 5:**
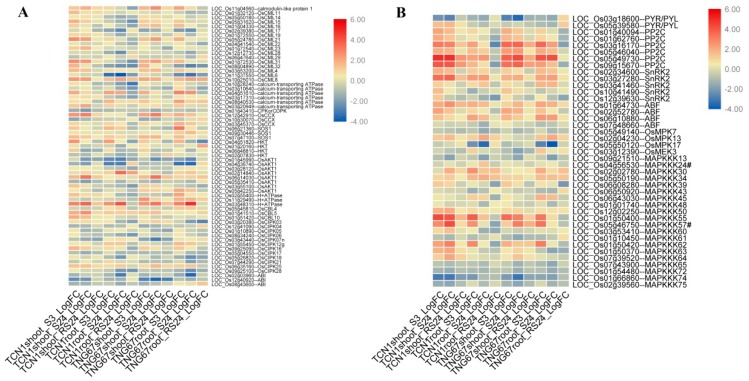
A heat map of the DEGs associated with the Ca^2+^ signal transduction pathway (**A**), the ABA signal transduction pathway, and the MAPK cascade (**B**).

The Ca^2+^ signal transduction pathway widely exists in eukaryotes [34]. Ca^2+^ acts as a second messenger through binding to Ca^2+^ sensors, causing a series of downstream reactions. At present, Ca^2+^ sensors can be divided into three types in plants: calmodulins (CAM/CML), calcium-dependent protein kinases (CDPKs), and calmodulins B-like proteins (CBLs) [35]. In this study, 63 DEGs related to the Ca^2+^ signaling pathway were identified, including genes encoding CMLs, CBLs, CDPKs, Ca^2+^-transporting ATPase, H^+^-ATPase, cation/Ca^2+^ exchangers (CCXs), SOS1, HKT, AKT1, calcineurin B-like–interacting protein kinases (CIPKs), and ABI (Figure 5A). Expression patterns revealed that three genes (*LOC_Os10g25010*, *LOC_Os06g14030*, and *LOC_Os01g41510*) were upregulated in shoots and roots of both genotypes at all time points and that three genes (*LOC_Os12g12730*, *LOC_Os01g43410*, and *LOC_Os01g45990*) were downregulated in shoots and roots of both genotypes at all time points. The remaining genes showed variable expression profiles under salt stress.

#### 3.5.2. DEGs Involved in the ABA Signal Transduction Pathway

In total, 17 DEGs associated with the ABA signaling transduction pathway were identified: two PYL genes, six PP2C genes, five SnRK2 genes, and four ABF genes (Figure 5B). The expression pattern results revealed that two PYL genes showed different expression patterns. *LOC_Os03g18600* was downregulated under salt stress, while *LOC_Os05g39580* was upregulated in TCN1 shoots under salt stress. Interestingly, these two genes both showed upregulation in TGN67 roots during the recovery process. Compared with PYL genes, the majority of PP2C genes showed upregulation in shoots and roots under salt stress. Similarly, the majority of SnRK2 and ABF genes were also upregulated in shoots and roots under salt stress.

#### 3.5.3. DEGs Involved in the MAPK Cascade Pathway

In this study, 24 DEGs related to the MAPK cascade pathway were identified: three MPK genes, one MEK gene, and 20 MAPKKK genes (Figure 5B). These genes showed unequal expression patterns. For example, *LOC_Os01g50400* (MAPKKK) was upregulated in shoots and roots under salt stress. However, *LOC_Os07g43900*, *LOC_Os01g54480*, *LOC_Os01g66860*, and *LOC_Os02g39560* showed downregulation at all tested points.

#### 3.5.4. Key TFs and Functional Proteins Related to Salt Stress

**Figure 6:**
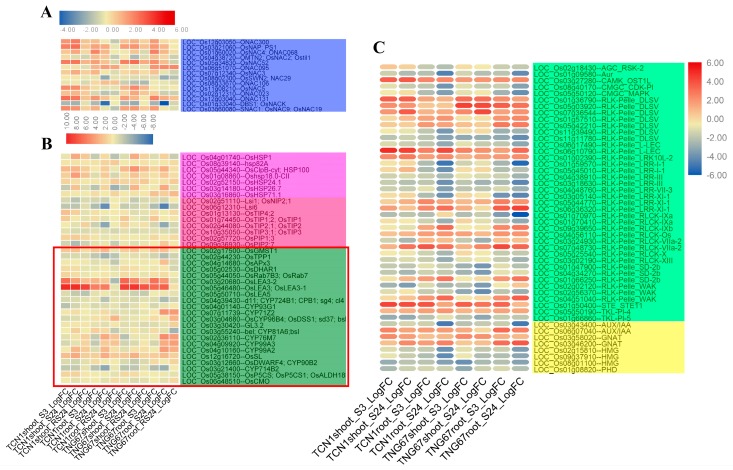
A heat map of NAC TFs of DEGs (**A**), functional protein genes of DEGs (**B**), and TR and PK genes of DEGs (**C**). Note: The genes in the red box are previously reported genes associated with salt stress.

We found that NAC (NAM, ATAF, and CUC) family genes formed the largest salt-responsive TF family of this study, and the majority of NAC family genes showed upregulation under salt stress (Figure 3 and Figure 6). Of these NAC genes, 14 have been reported in previous studies (Figure 6A) and their functions were associated with plant senescence delay [36], abiotic stress responses [37], cellulose synthesis [38], and others [39,40]. These results imply that the NAC family is a multifunctional family and also plays vital roles in regulating plant salt stress tolerance. Thus, NAC family genes may be good candidate genes in salt stress tolerance for genetic engineering breeding.

Previous reports have indicated that many functional proteins, including AQPs (aquaporins), HSPs (heat shock proteins), LEA (late embryogenesis abundant) proteins, F-box proteins, transporter proteins, and other functional proteins are differentially expressed under salt stress and play key roles in regulating plant salt tolerance [24,41]. In this study, seven HSPs and eight AQPs were identified in DEGs (Figure 6B). In addition, 22 of the previously identified functional proteins also were found in this study’s DEGs.

#### 3.5.5. TRs and PKs Involved in Salt Stress

TRs and PKs play vital roles in rice tolerance to abiotic stresses [24]. However, they have been rarely mentioned in previous salt stress studies. In this study, many TRs and PKs were differentially expressed under salt stress (Figure 6C). For instance, one-third of the PK genes showed upregulation under salt stress. In TRs, AUX/IAA and GNAT genes showed upregulation under salt stress, while HMG and PHD genes were downregulated under salt stress.

### 3.6. Key Genes Associated with Higher Salt Stress Tolerance in TNG67

**Figure 7:**
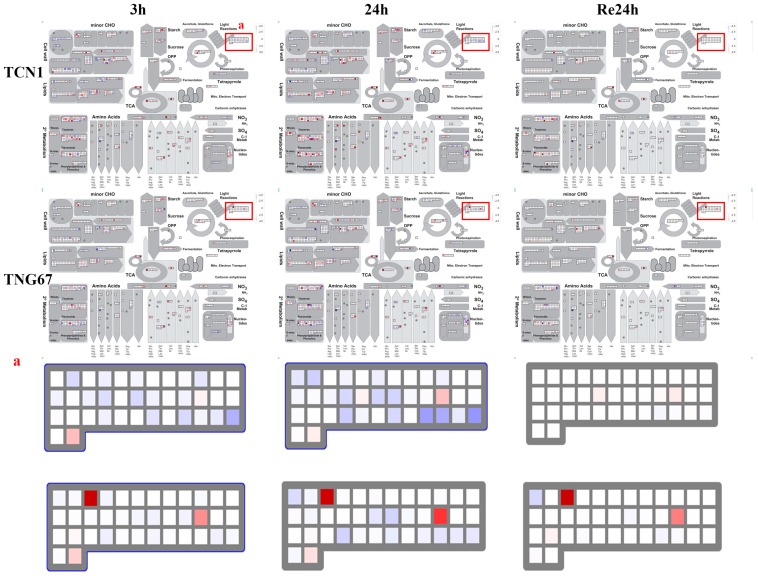
The MapMan analysis of all DEGs involved in the metabolism overview in shoots at 3 h and 24 h after salt stress treatment and at a subsequent 24-h recovery point. The excised panel (**a**) represents genes associated with light reactions. A high resolution version of Figure 7 is provided in Figure S7.

**Figure 8:**
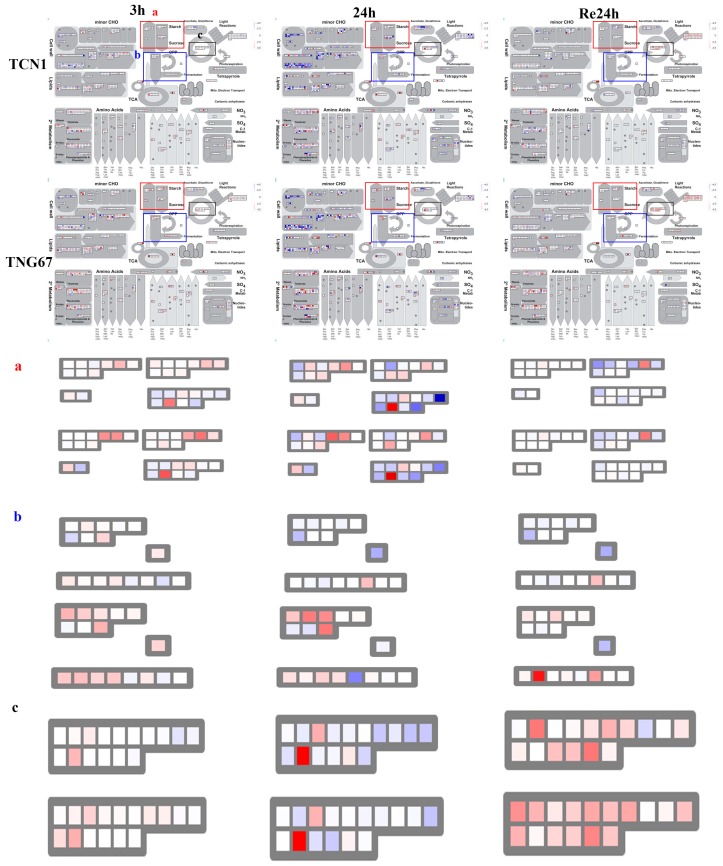
The MapMan analysis of all DEGs involved in the metabolism overview in roots at 3 h and 24 h after salt stress treatment and at a subsequent 24-h recovery point. The excised panel (**a**) represents genes associated with starch and sucrose. The excised panel (**b**) represents genes associated with glycolysis and fermentation. The excised panel (**c**) represents genes associated with the Calvin cycle. A high resolution version of Figure 8 is provided in Figure S8.

To investigate the possible reason for different salt stress tolerances between TCN1 and TNG67, metabolic pathways were visualized by a metabolism overview in MapMan 3.6.0. For shoots, these results revealed a greater enhancement of gene expression associated with light reactions in TNG67 than in TCN1 under salt stress (Figure 7). For roots, there was a greater enhancement of gene expression associated with starch, sucrose, and fermentation in TNG67 than in TCN1 under salt stress (Figure 8). Interestingly, we observed that Calvin cycle genes had higher expression levels in TNG67 than in TCN1 during the recovery process (Figure 8). Thus, we speculated that TNG67 shifted to a fermentation pathway to produce energy for growth under salt stress and produced lots of starch and sucrose for coping with osmotic stress. In addition, TNG67 may enhance the Calvin cycle to repair a damaged photosystem during the recovery stage.

## 4. Discussion

### 4.1. The Ca^2+^ Signaling Pathway, ABA Signaling Transduction Pathway, and MAPK Cascade Pathway Play Important Roles under Salt Stress Conditions

Previous genetic and biochemical studies have revealed that many genes participate in salt resistance via hormone and Ca^2+^ signal pathways, TFs, ion metabolism, ion transfer, nitrogen metabolism, and secondary metabolism [3,8,24]. In this study, these genes were also identified. A total of 63, 17, and 24 DEGs were identified in Ca^2+^ signaling pathway, the ABA signaling transduction pathway, and the MAPK cascade pathway, respectively. These results support previous findings that these pathways can form a close-knit signaling network and play vital roles in plant salt stress tolerance by interacting with each other or starting up downstream factors, such as TFs, TRs, and functional protein genes (Figure 3) [41,42,43].

### 4.2. Many TFs and PKs May Be Good Candidate Genes for Plant Salt Stress Tolerance Breeding

Previous studies have reported that NAC, MYB, bHLH, and AP2/ERF are associated with salt stress tolerance [44]. Of these TFs, some TFs are associated with stress-signal pathways. For example, Zhang et al. (2017) reported that several bHLH-TFs are associated with the ABA signal pathway [44]. Jakoby et al. (2002) reported that bZIP-TFs are downstream factors of the ABA signal pathway and regulate the pivotal cell process in the response to salt stress [45]. Zhang et al. (2012) reported that HD-Zip-TFs affect ABA biosynthesis and regulate rice salt tolerance via the ABA signal pathway [46]. Similarly, we also found that TFs participate in the plant stress response via the ABA signal pathway. In this study, we found that *LOC_Os02g17500* (encoding a GRAS TF) was associated with SnRK (*LOC_Os03g27280*, key genes for the ABA signal pathway). However, beyond that, TFs are also regulators of salt stress. For example, Jiang et al. (2017) reported that WRKY-TFs are important regulators for salt stress tolerance [47]. OsMYB2 is an important regulator for salt stress in rice [48]. Some previously reported TFs (associated with abiotic stress) were found to be DEG-TFs in this study. These genes may also play vital roles in the salt stress tolerance of rice, such as *LOC_Os03g48780*, *LOC_Os05g49730*, *LOC_Os04g56430*, *LOC_Os03g48750*, and *LOC_Os09g31031* (Table S7). For instance, several reported TFs in salt stress were present in the DEG-TFs identified in this study, such as Oshox22 (*LOC_Os04g45810*, HD-ZIP) [46] and OsHsfB2b (*LOC_Os08g43334*, HSP) [49]. In this study, many TFs were identified and can be important candidate genes for salt stress breeding. Among them, two HD-ZIP genes (*LOC_Os06g46740* and *LOC_Os02g43330*) showed high upregulation (LogFC >2.0, Table S7). Similarly, two MYB genes (*LOC_Os01g54030* and *LOC_Os08g39730*) also were highly upregulated (LogFC >3.0, Table S7). These genes can be important for rice salt stress tolerance.

RLKs (receptor-like protein kinases), one of the largest gene families in plants, play vital roles in the regulation of plant developmental processes, signaling networks, and disease resistance [50]. Previous studies have reported that many RLKs have been proved to be involved in abiotic stress responses, including the ABA response, Ca^2+^ signaling, and antioxidant defense [50]. Vaid et al. (2015) reported that *Pisum sativum* LecRLKs (*PsLecRLKs*) were upregulated under salt stress and *PsLecRLK*-overexpressing plants had a greater tolerance to salt stress than wild-type plants due to ROS-scavenging enzymes, reducing ROS accumulation and leading to lower membrane damage [51]. Li and Sun (2014) found that *SIT1*, a *LecRLK* gene mainly expressed in root epidermal cells, mediated rice salt sensitivity [52]. In this study, many TR genes were upregulated under salt stress. RLKs may be good candidate genes for rice salt stress tolerance breeding.

### 4.3. Common DEGs between This Study and Previous Studies Contain Important Salt-Responsive Genes

**Figure 9:**
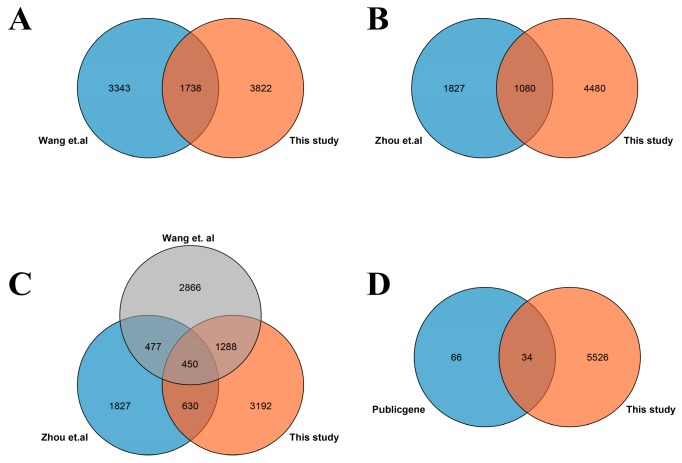
A Venn diagram of the DEGs of this study with those of previous studies, including Wang et al. (**A**) [8], Zhou et al. (**B**) [23], Wang et al. and Zhou et al. (**C**), and functionally characterized genes (**D**).

Common DEGs from multiple salt stress studies can help us to identify core salt-responsive genes. In this study, we conducted a comparative analysis of this study with two previous salt stress studies. This result showed that a total of 1738 and 1080 common DEGs were found with the Wang et al. study [8] and the Zhou et al. study [23], respectively (Figure 9A,B, Tables S8 and S9). Next, 450 common DEGs were found in these three studies (Figure 9C). Based on MSU7.0 description and expression data, we noticed that several genes may be good candidate genes for future salt stress tolerance breeding. For example, *LOC_Os01g11730* (a GDSL-like lipase/acylhydrolase gene), *LOC_Os05g31670* (encoding AWPM-19-like membrane family protein), *LOC_Os01g21420* (encoding pre-mRNA-splicing factor, SF2), and *LOC_Os05g31020* (encoding eukaryotic peptide chain release factor subunit 1-1) showed significant upregulation under salt stress (Table S10). Moreover, we found that 100 salt-responsive genes were reported in previous studies (Table S11). Of these genes, 34 were identified as DEGs in this study (Table S12). This result revealed that many unreported salt-responsive genes were found in this study.

### 4.4. Multiple DEGs Mapped to the Previously Identified Salt-Stress-Related QTL Intervals and Could Be Good Candidate Genes for Rice Salt Stress Tolerance Breeding

**Figure 10:**
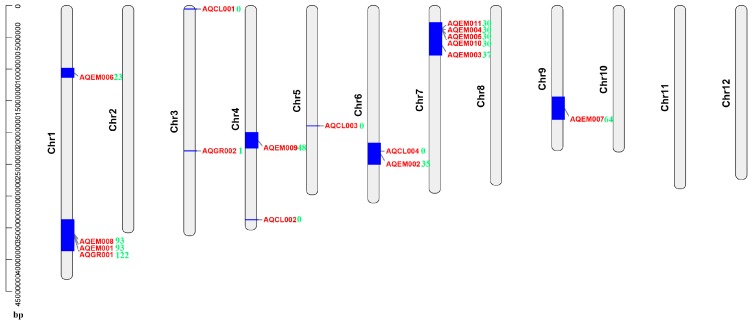
Co-localization of DEGs onto the previously detected quantitative trait loci (QTLs) responsible for salt treatment in rice. Red words denote previously detected QTLs, green numbers denote the DEGs of this study on previously detected QTLs.

The previously identified salt-stress-related QTLs can help us to target candidate genes. A total of 17 QTLs were obtained from the Gramene QTL database [23]. The DEGs of this study were mapped to 12 QTLs (Table S13). Among them, AQGR001, AQEM001, AQEM008, and AQEM007 had the greatest number of co-localized DEGs: 122, 93, 93, and 64, respectively (Figure 10). A previous study reported that AQEM002 and AQGR001 are the most important QTLs related to rice salt stress tolerance at the seedling stage [12,53]. In this study, 35 and 122 DEGs, respectively, were co-localized on these two QTLs. These DEGs could be good candidate genes for rice salt stress tolerance breeding, such as *LOC_Os01g64360* (one MYB gene, in AQGR001), *LOC_Os01g64790* (one AP2/ERF gene, in AQGR001), *LOC_Os01g62760* (one PP2C gene, in AQGR001), *LOC_Os06g39040* (one dehydrogenase/reductase SDR family gene). Interestingly, only one gene (*LOC_Os03g41064*) was mapped on AQGR002, which encodes one natural-resistance-associated macrophage protein. Thus, *LOC_Os03g41064* may play a vital role under salt stress.

## Figures and Tables

**Table 1:** The biological process categories of Gene Ontology (GO) annotation of core salt-responsive genes in shoots under salt stress. The GO enrichment analysis of core salt-responsive genes was implemented by the GOseq R packages, and GO terms with *p* < 0.001 were adopted in this study. Red terms represent common terms of shoots and roots.

Gene_Ontology_term	Cluter_frequency	*p*-Value
heat acclimation (GO:0010286);	5.70%	0
response to cadmium ion (GO:0046686);	14.25%	4.16E-10
response to water deprivation (GO:0009414);	14.81%	1.59E-09
response to cold (GO:0009409);	13.11%	2.01E-09
response to salt stress (GO:0009651);	14.53%	8.65E-08
positive regulation of transcription, DNA-templated (GO:0045893);	10.83%	9.72E-08
response to heat (GO:0009408);	7.12%	1.66E-07
hyperosmotic salinity response (GO:0042538);	7.41%	3.62E-07
response to high light intensity (GO:0009644);	6.27%	4.43E-07
response to abscisic acid (GO:0009737);	11.68%	3.43E-06
negative regulation of abscisic acid-activated signaling pathway (GO:0009788);	3.70%	5.57E-06
response to hydrogen peroxide (GO:0042542);	5.70%	0.0000161
negative regulation of seed dormancy process (GO:1902039);	1.14%	0.0001145
leaf senescence (GO:0010150);	4.84%	0.0001683
negative regulation of protein kinase activity (GO:0006469);	1.42%	0.0002851
toxin catabolic process (GO:0009407);	4.56%	0.0005736
cell proliferation (GO:0008283);	4.00%	0.0010226
negative regulation of transcription, DNA-templated (GO:0045892);	4.84%	0.0018958
response to chitin (GO:0010200);	8.55%	0.0022302
response to hypoxia (GO:0001666);	2.85%	0.0038614
response to organic substance (GO:0010033);	6.27%	0.0043247
response to wounding (GO:0009611);	8.26%	0.0045121
protein folding (GO:0006457);	4.84%	0.0050309
release of seed from dormancy (GO:0048838);	1.14%	0.005182
hydrogen peroxide catabolic process (GO:0042744);	3.13%	0.0056712
glucose catabolic process (GO:0006007);	3.42%	0.0062763
ethylene biosynthetic process (GO:0009693);	3.13%	0.007078
ethylene-activated signaling pathway (GO:0009873);	3.99%	0.0072325
PSII associated light-harvesting complex II catabolic process (GO:0010304);	2.00%	0.0081537
photoinhibition (GO:0010205);	1.42%	0.0083607

Note: A total of 351 core salt-responsive genes in shoots were enriched on GO terms. Cluter_frequency = enriched gene numbers of each term/351.

**Table 2:** The biological process categories of Gene Ontology (GO) annotation of core salt-responsive genes in roots under salt stress. The GO enrichment analysis of core salt-responsive genes was implemented by the GOseq R packages, and GO terms with *p* < 0.001 were adopted in this study. Red terms represent common terms of shoots and roots.

Gene_Ontology_term	Cluter_frequency	*p*-Value
hyperosmotic salinity response (GO:0042538);	8.39%	0
response to cold (GO:0009409);	13.12%	0
response to cadmium ion (GO:0046686);	13.12%	0
response to salt stress (GO:0009651);	15.05%	0
response to oxidative stress (GO:0006979);	7.74%	0
response to nitrate (GO:0010167);	7.10%	2.164E-10
defense response to fungus (GO:0050832);	10.97%	5.793E-10
response to desiccation (GO:0009269);	4.30%	8.921E-10
nitrate transport (GO:0015706);	7.10%	1.275E-09
salicylic acid mediated signaling pathway (GO:0009863);	5.16%	2.674E-08
response to water deprivation (GO:0009414);	10.54%	3.62E-08
root hair elongation (GO:0048767);	6.88%	4.066E-08
response to abscisic acid (GO:0009737);	10.98%	4.342E-07
lateral root morphogenesis (GO:0010102);	2.80%	7.002E-07
oxidation-reduction process (GO:0055114);	12.69%	2.418E-06
response to auxin (GO:0009733);	7.31%	5.595E-06
defense response to nematode (GO:0002215);	1.94%	6.042E-06
oligopeptide transport (GO:0006857);	3.87%	2.173E-05
response to cyclopentenone (GO:0010583);	3.87%	2.421E-05
response to wounding (GO:0009611);	8.60%	2.649E-05
lipid transport (GO:0006869);	2.79%	0.0002419
plant-type cell wall organization (GO:0009664);	5.38%	0.0002581
transition metal ion transport (GO:0000041);	3.44%	0.0003009
selenate transport (GO:0080160);	1.075%	0.0005686
lignin biosynthetic process (GO:0009809);	3.23%	0.000737

Note: A total of 465 core salt-responsive genes in roots were enriched on GO terms. Cluter_frequency = enriched gene numbers of each term/465.

## Supplementary Materials

The following are available online at https://www.mdpi.com/2223-7747/8/3/64/s1, Figure S1: Venn diagram of salt-responsive DEGs in shoots and in roots. Figure S2: Heat map of salt-responsive genes (123 common DEGs in shoots and roots in both genotypes). Figure S3: Heat map of salt-responsive genes in shoots (254 common DEGs in shoots in both genotypes). Figure S4: Heat map of salt-responsive genes in roots (365 common DEGs in shoots in both genotypes). Figure S5: qRT-PCR results of DEGs in ‘Nipponbare’. Figure S6: MapMan biotic stress overview of core salt-responsive genes (742 DEGs) in shoots after 3 h, 24 h, and recovery 24 h in shoots, as well as 3 h, 24 h, and recovery 24 h in roots. Table S1: Detailed information of GSE76613. Table S2: Primers of qRT-PCR for DEG validation. Table S3: All expression datasets of DEGs under salt and recovery conditions in shoots and roots of the two genotypes. Table S4: Expression datasets of Figure S2 DEGs. Table S5: Expression datasets of Figure S3 DEGs. Table S6: Expression datasets of Figure S4 DEGs. Table S7: Expression datasets of TF genes (these genes are identified in 742 core salt-responsive genes). Table S8: Common DEGs between this study and the Wang et al. study. Table S9: Common DEGs between this study and the Zhou et al. study. Table S10: Common DEGs of this study, the Wang et al. study, and the Zhou et al. study. Table S11: Details of previously reported salt responsive genes (100 genes). Table S12: The common genes between this study and previously reported salt responsive genes. Table S13. Co-localization of DEGs onto the previously detected QTLs responsible for salt treatment in rice.
