# Supplementary material for: Meta-Analysis of Salt Stress Transcriptome Responses in Different Rice Genotypes at the Seedling Stage
Source: Plants (Basel). 2019 Mar 12;8(3):64. doi: 10.3390/plants8030064 (PMC6473595; doi:10.3390/plants8030064)
Supplement: Supplementary file 1 [file plants-08-00064-s001.zip › sup/Fig S5.pdf]

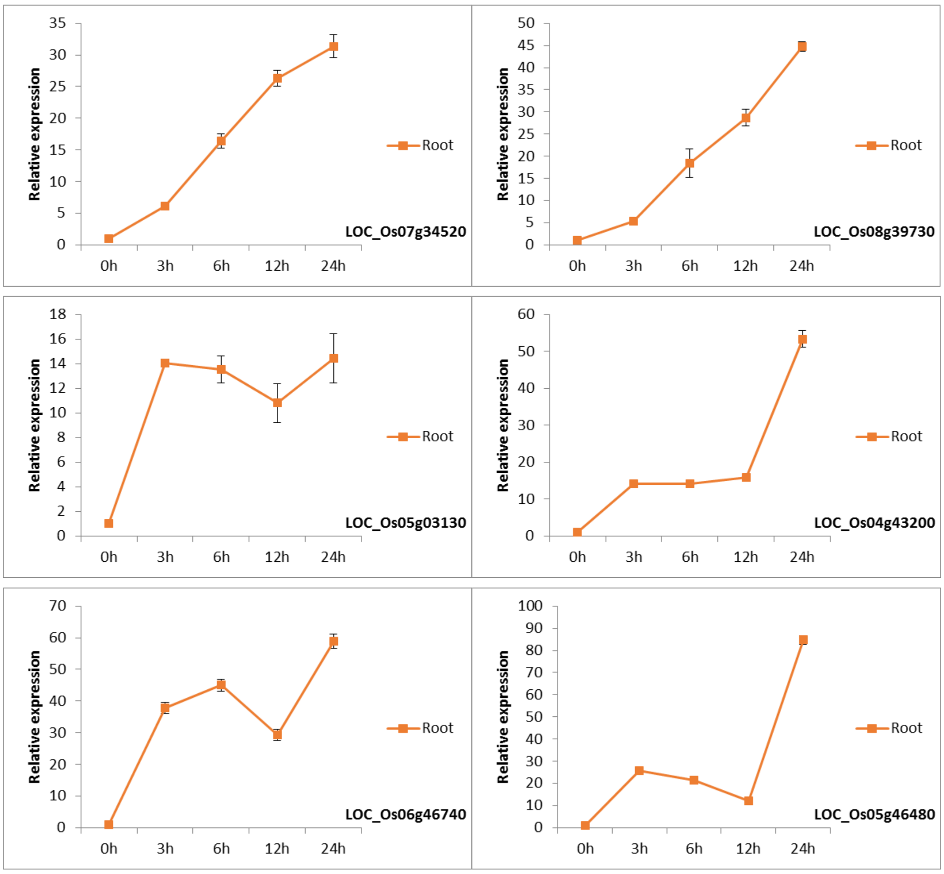

Expression pattern of the 6 DEGs in ‘Nipponbare’ seedling roots after NaCl treatment 0h, 3h, 6h, 12h, and 24h. The $2^{-\Delta\Delta CT}$ method is adopted to calculate the fold change of DEGs expression from three biological replicates.
