# Supplementary figures and images for: Meta-Analysis of Salt Stress Transcriptome Responses in Different Rice Genotypes at the Seedling Stage
Source: Plants (Basel). 2019 Mar 12;8(3):64. doi: 10.3390/plants8030064 (PMC6473595; doi:10.3390/plants8030064)

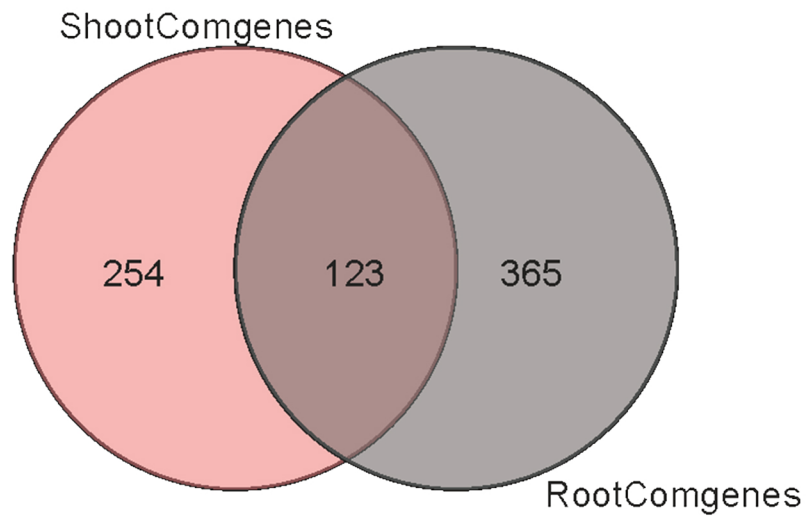

Supplement: Supplementary file 1 [file plants-08-00064-s001.zip › sup/Fig S1.pdf]

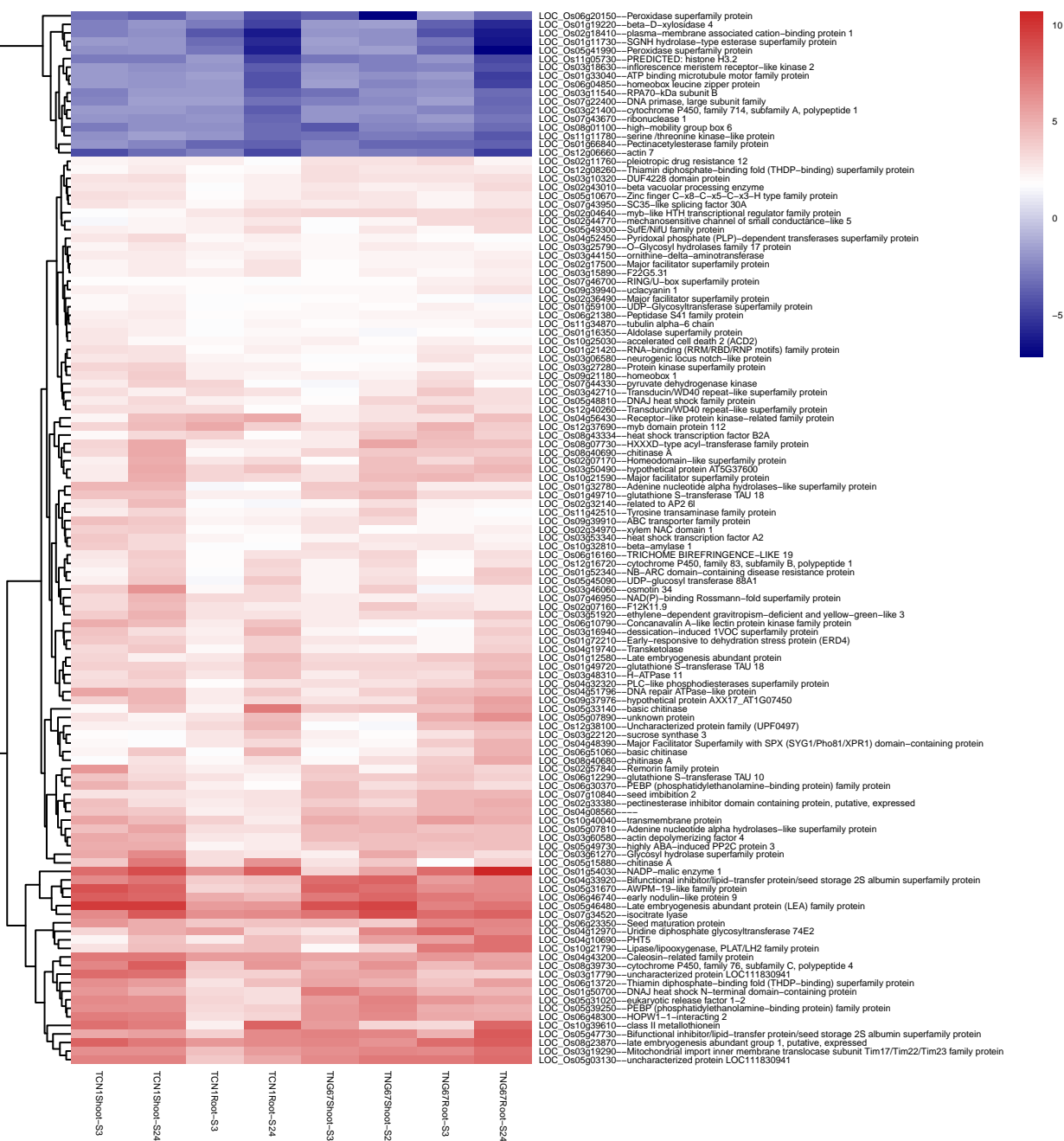

Supplement: Supplementary file 1 [file plants-08-00064-s001.zip › sup/Fig S2.pdf]

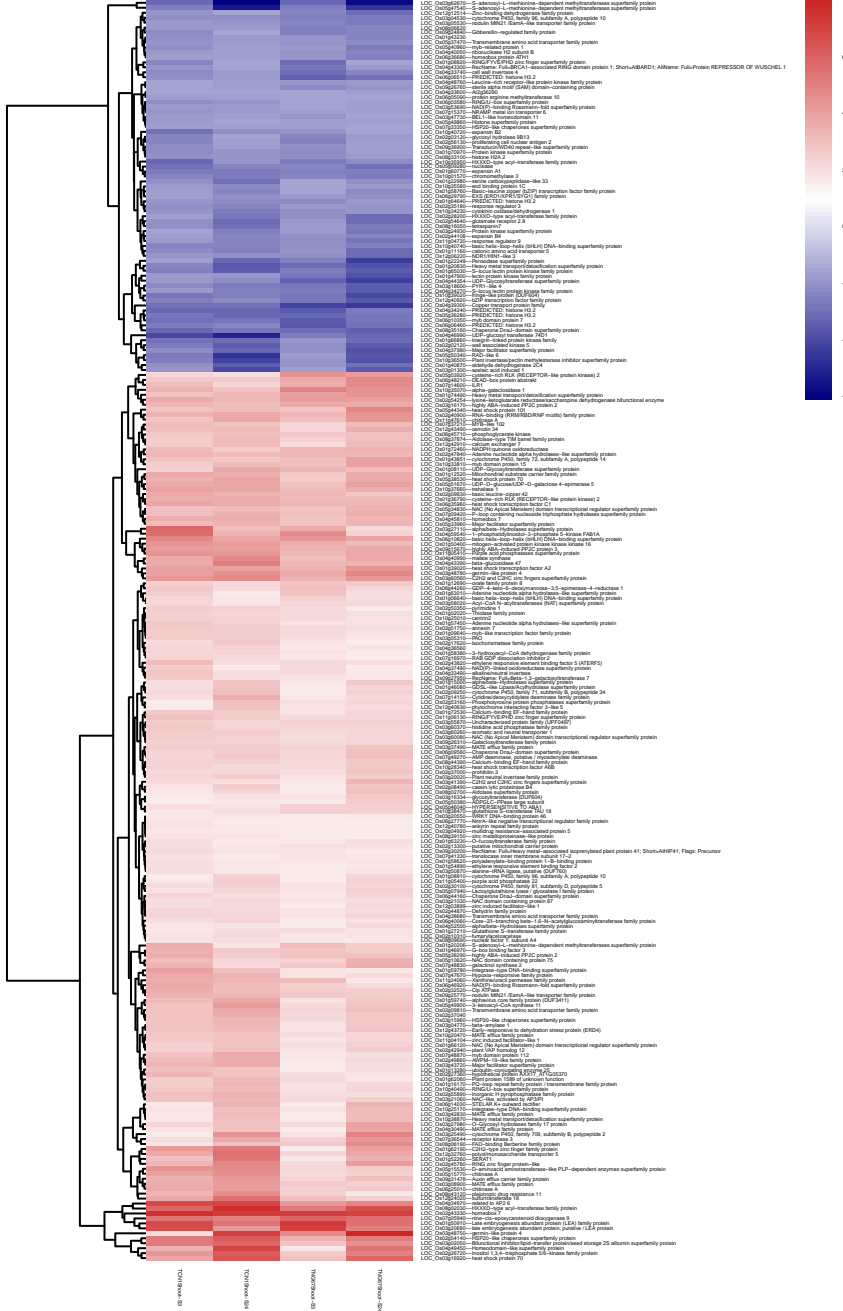

100% WT

100% WT+L2

100% WT+L3

100% WT+L4

6  
4  
2  
0  
-2  
-4  
-6

Supplement: Supplementary file 1 [file plants-08-00064-s001.zip › sup/Fig S3.pdf]

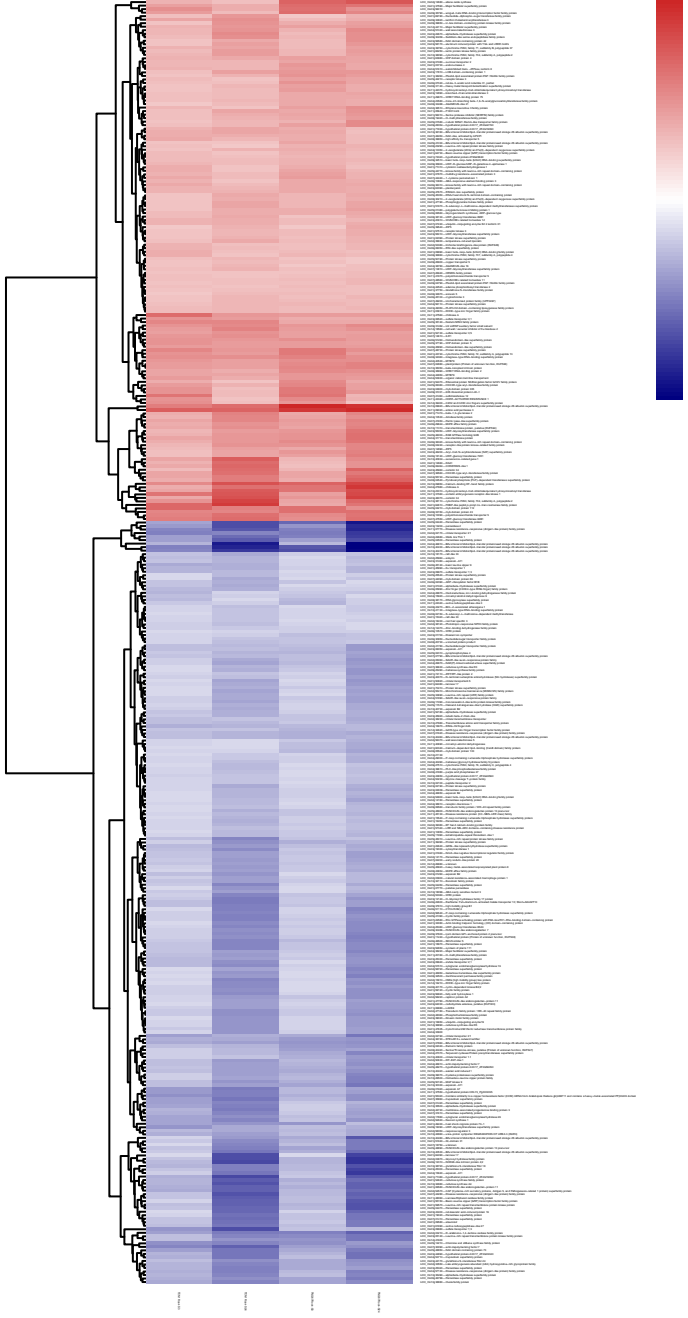

1000000000

1000000000

1000000000

1000000000

Supplement: Supplementary file 1 [file plants-08-00064-s001.zip › sup/Fig S4.pdf]

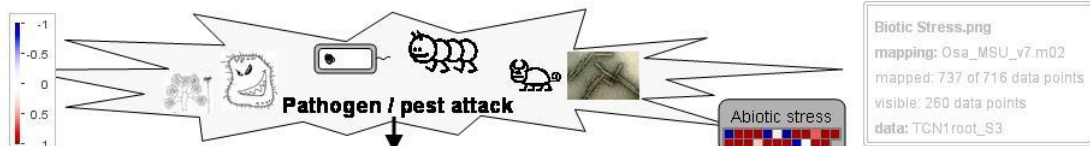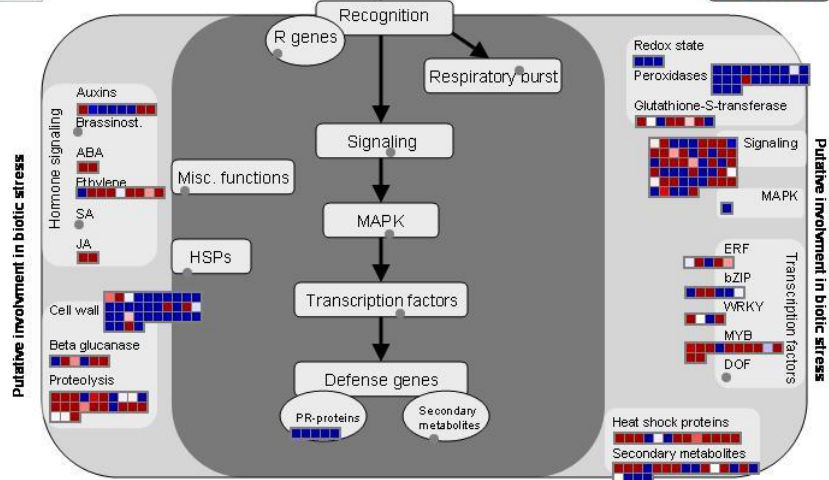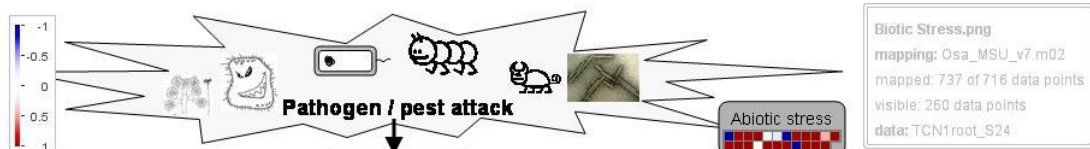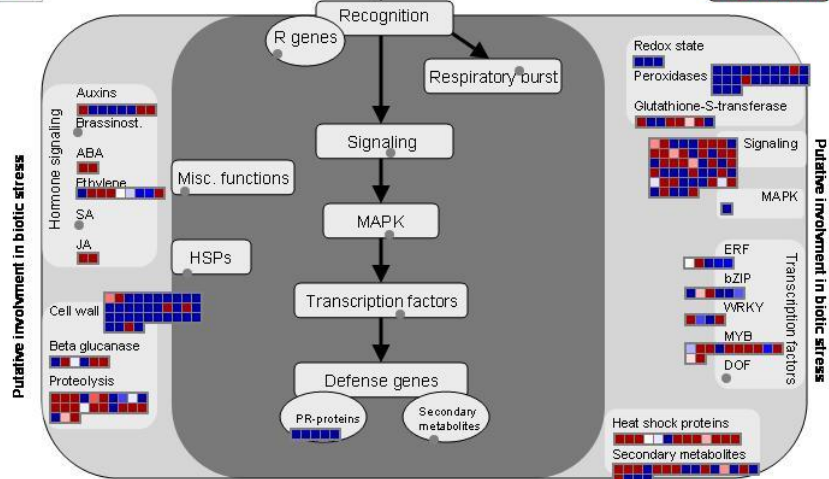

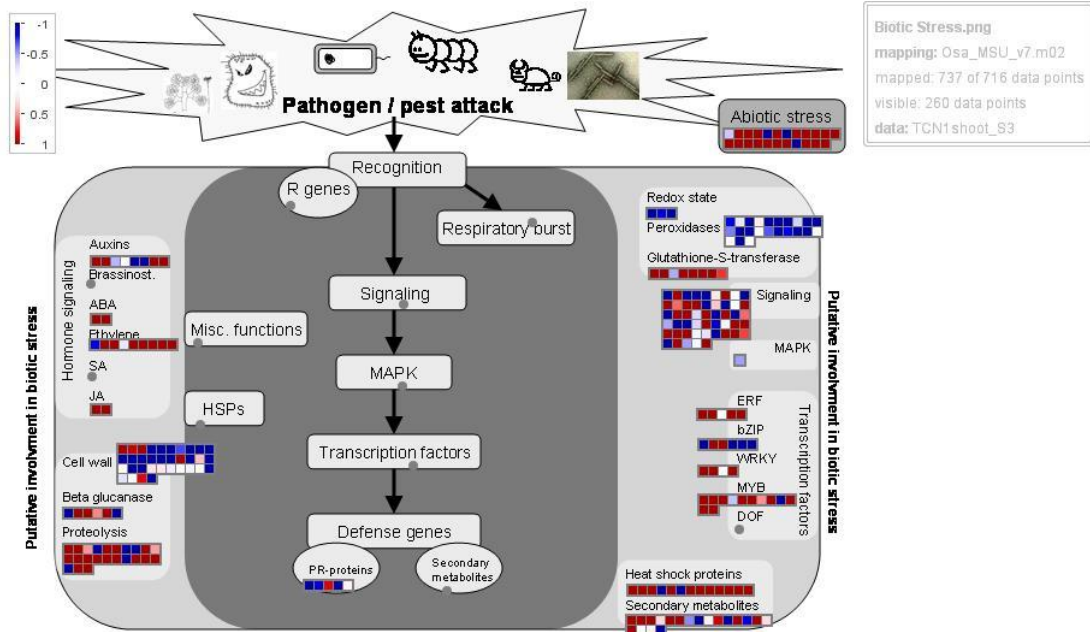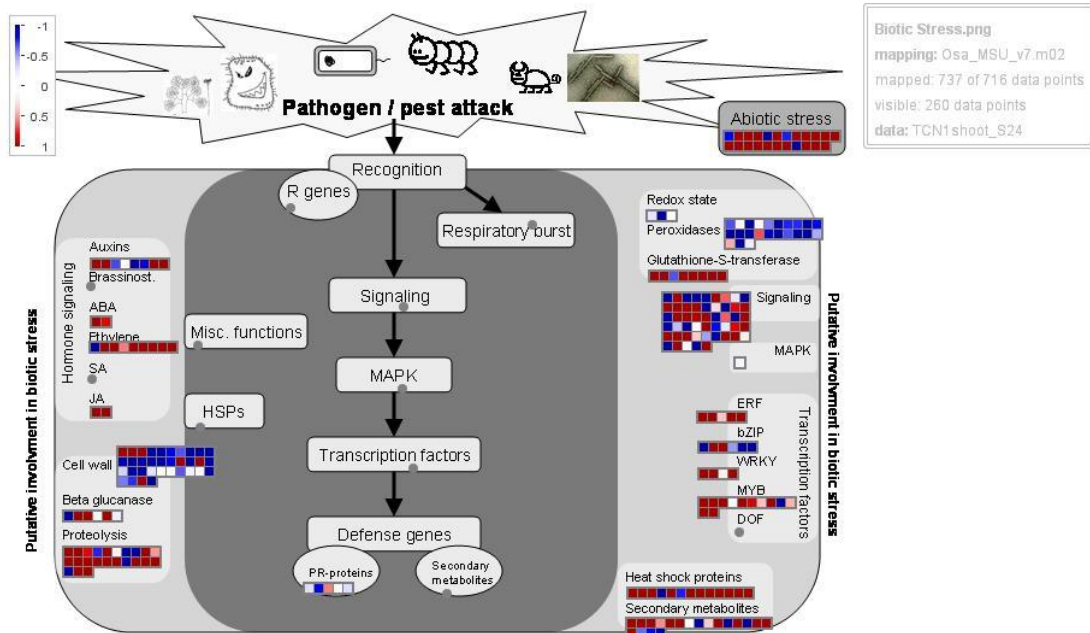

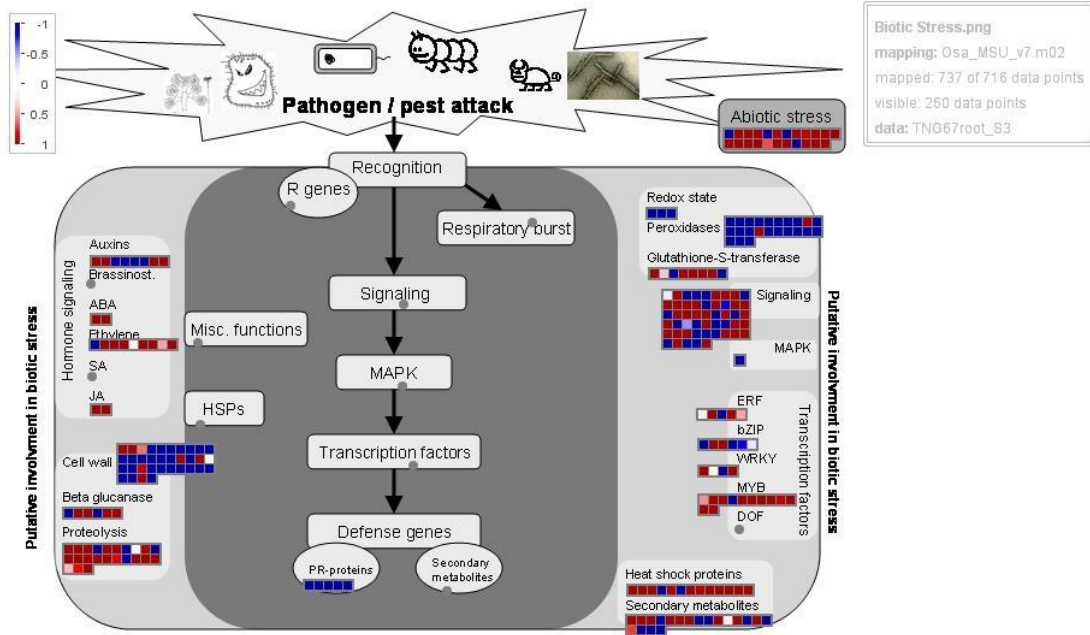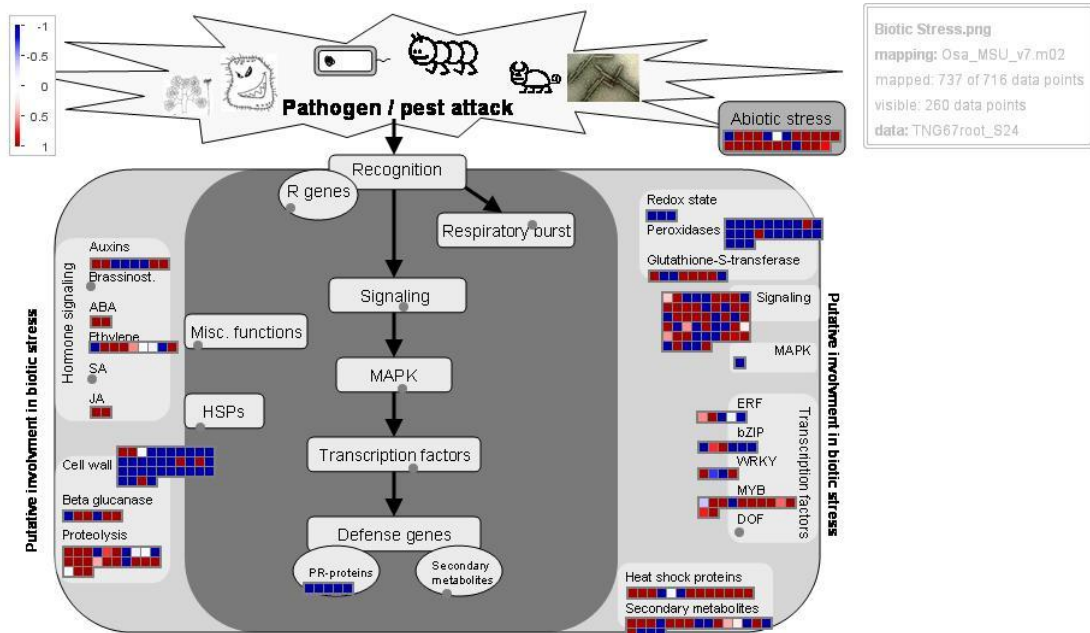

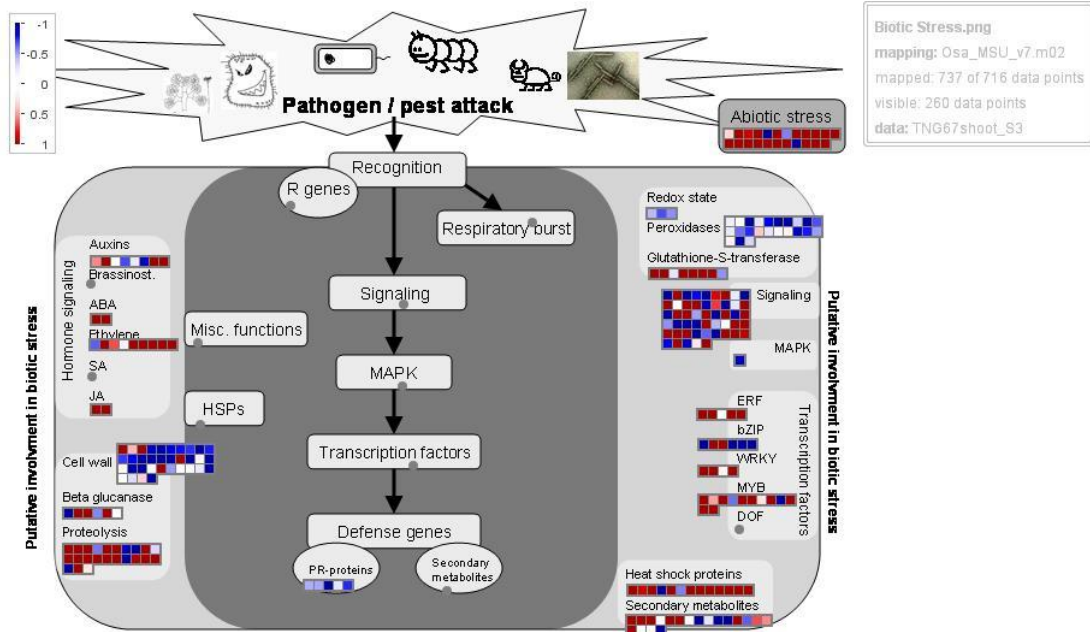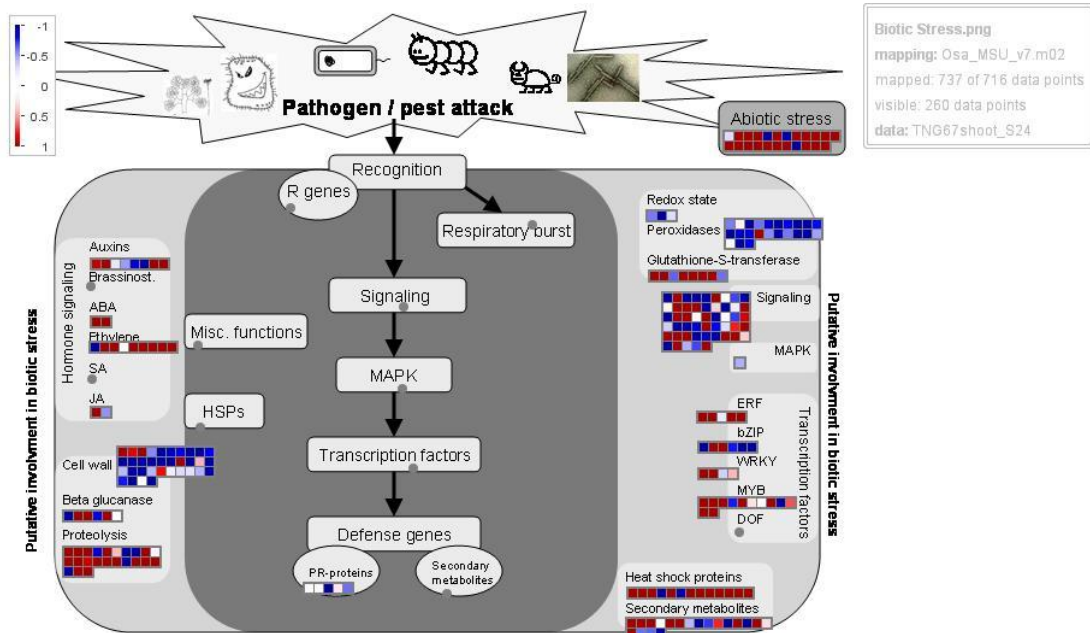

Supplement: Supplementary file 1 [file plants-08-00064-s001.zip › sup/Fig S6.pdf]

TCN1

3h

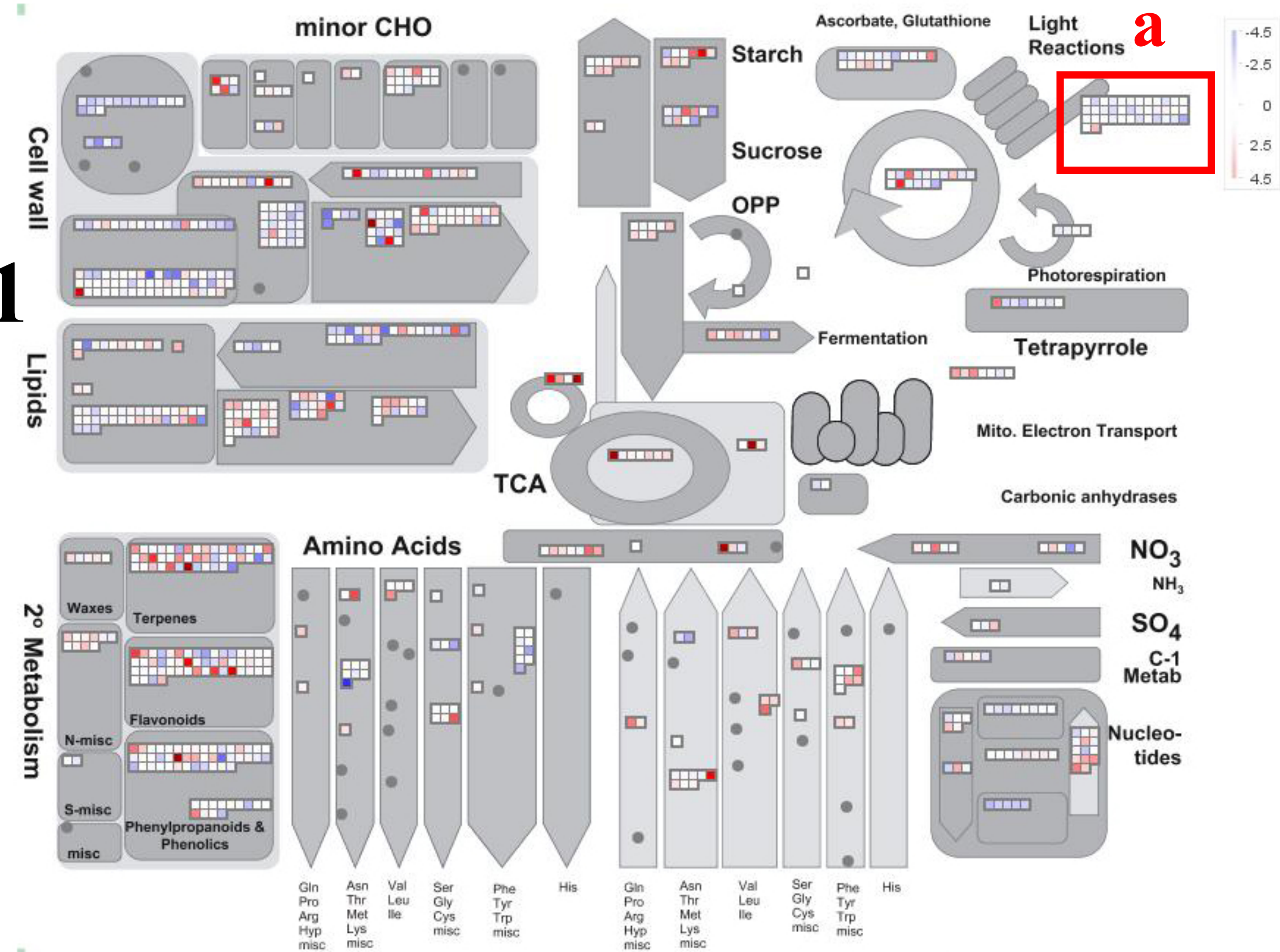

24h

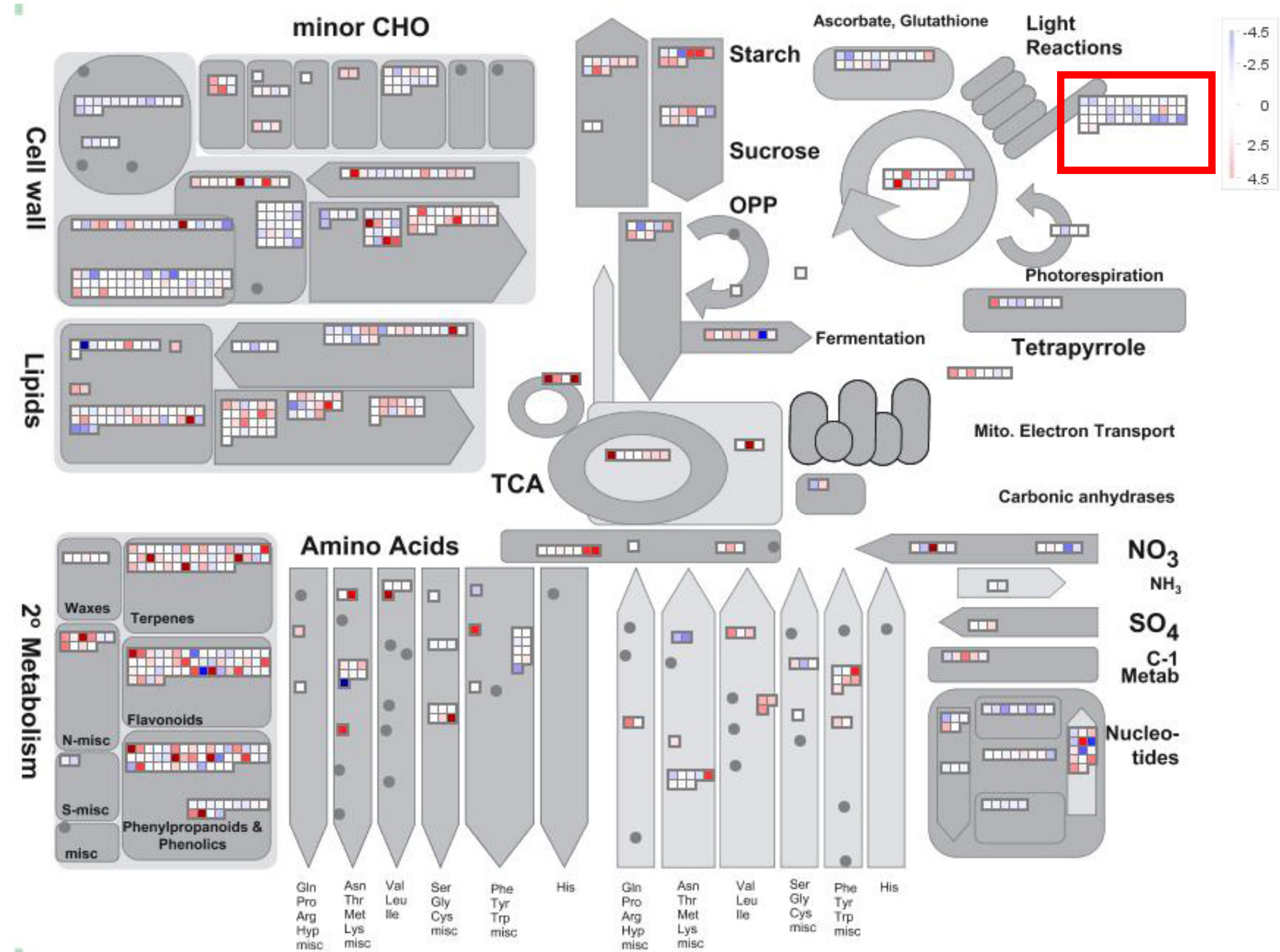

Re24h

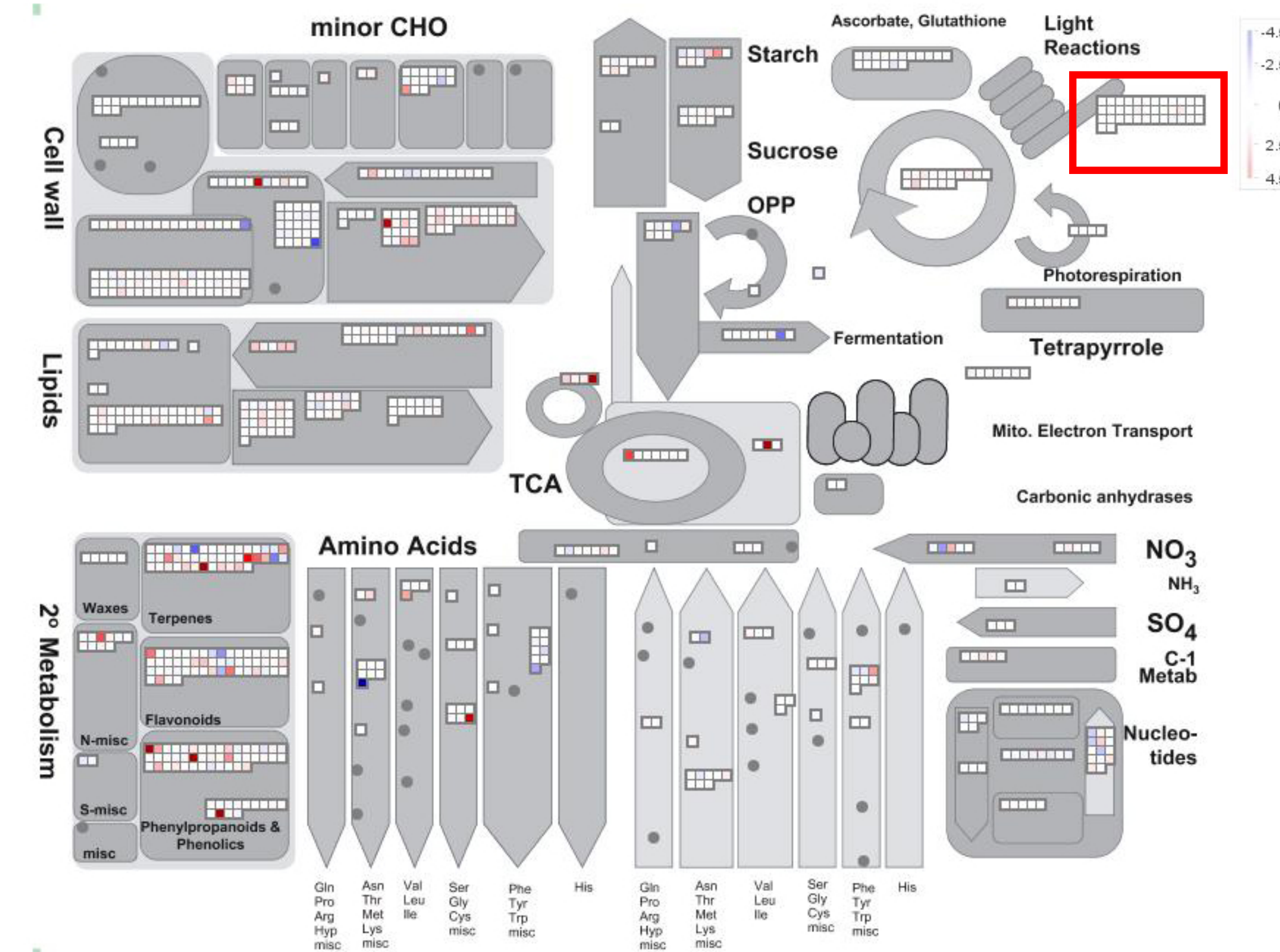

TNG67

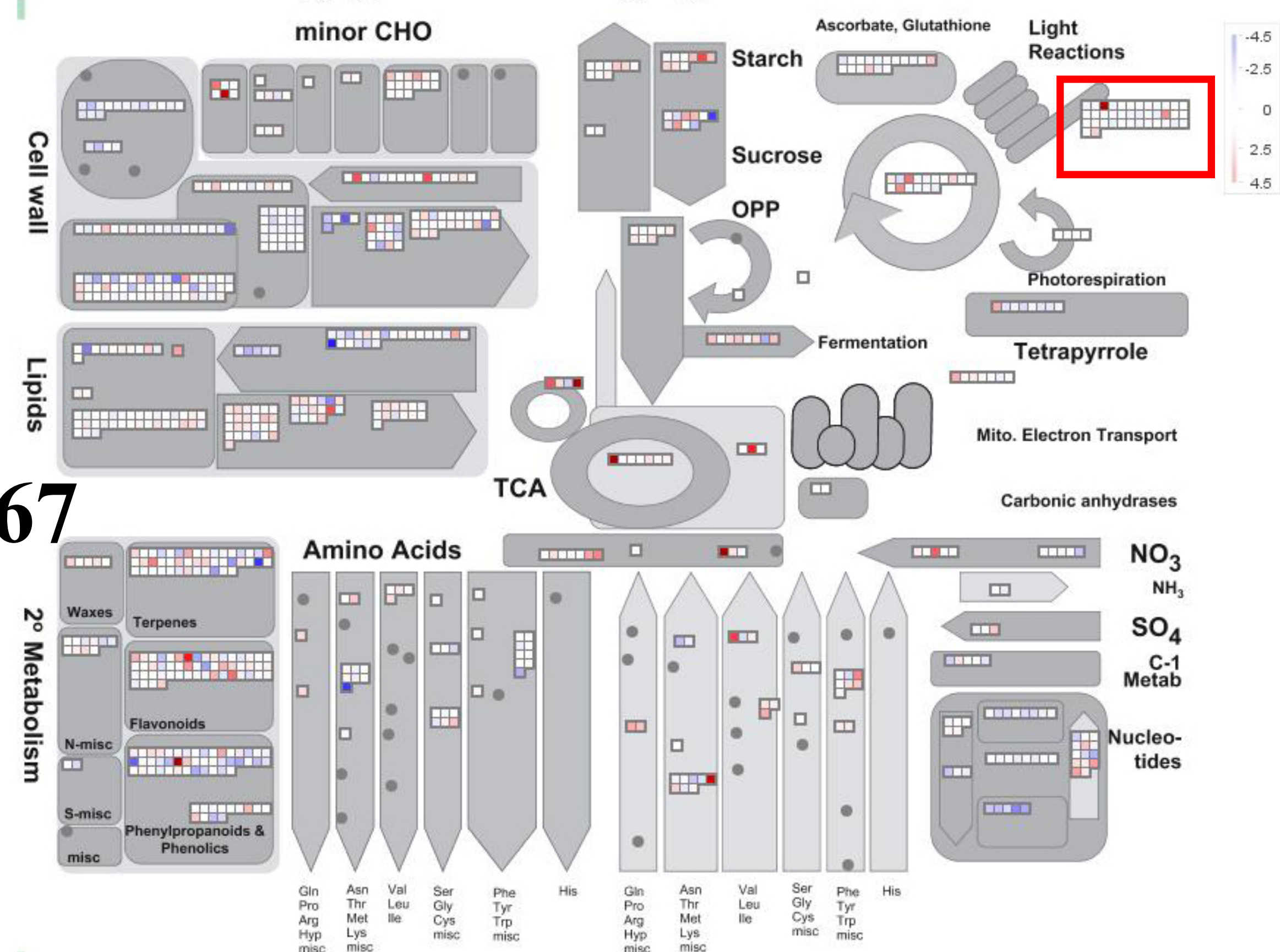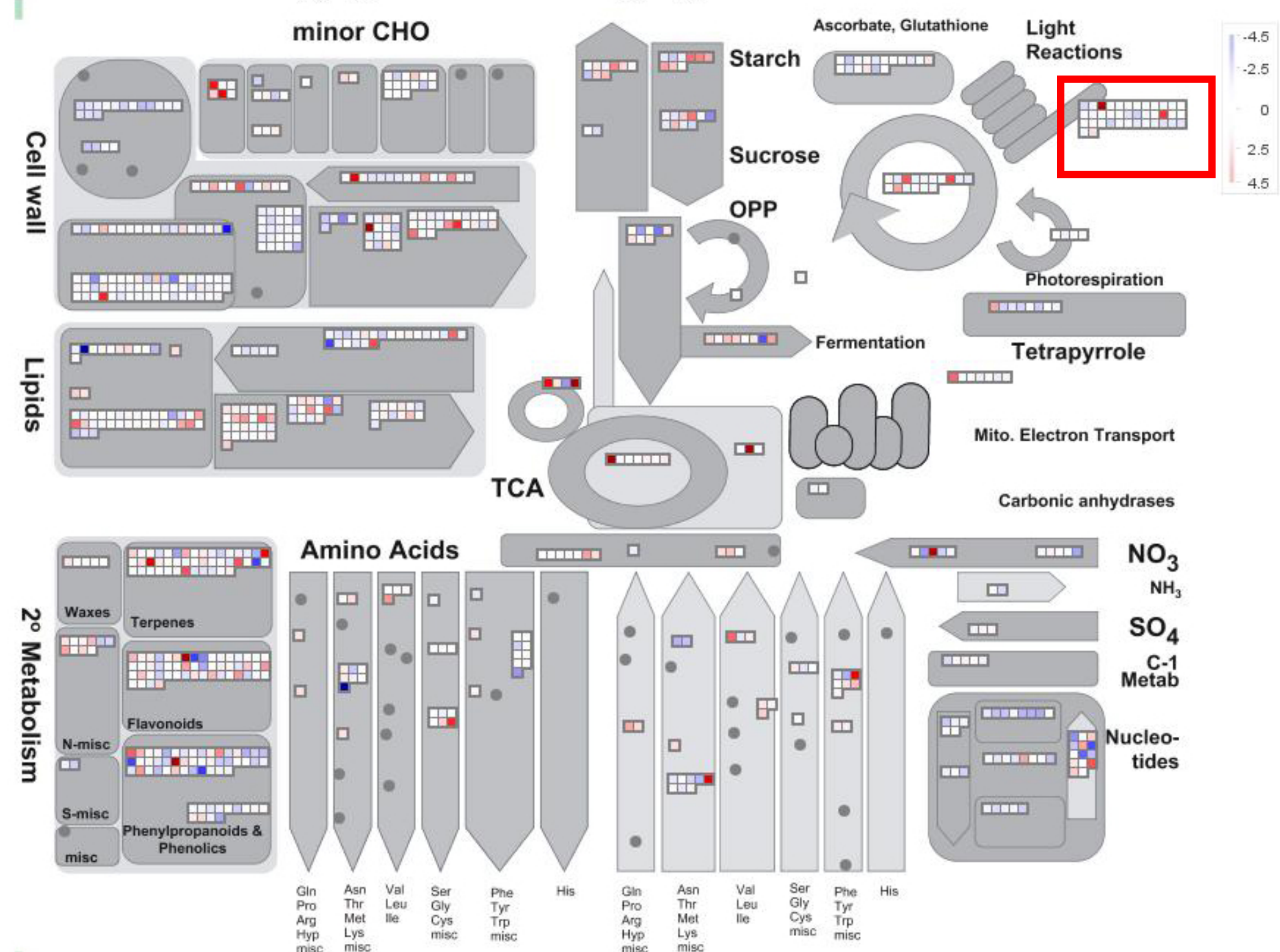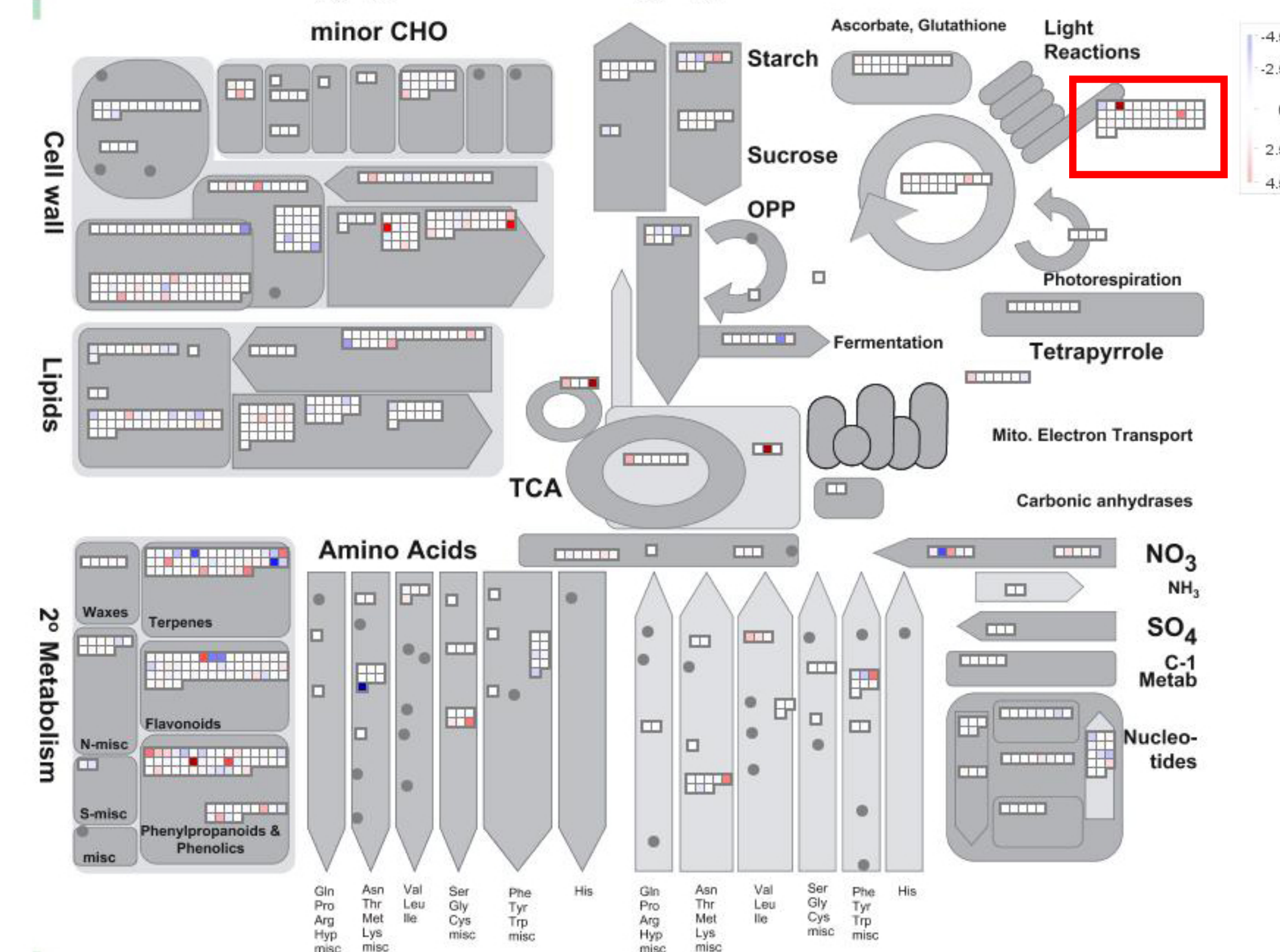

Supplement: Supplementary file 1 [file plants-08-00064-s001.zip › sup/Fig S7.pdf]

TCN1

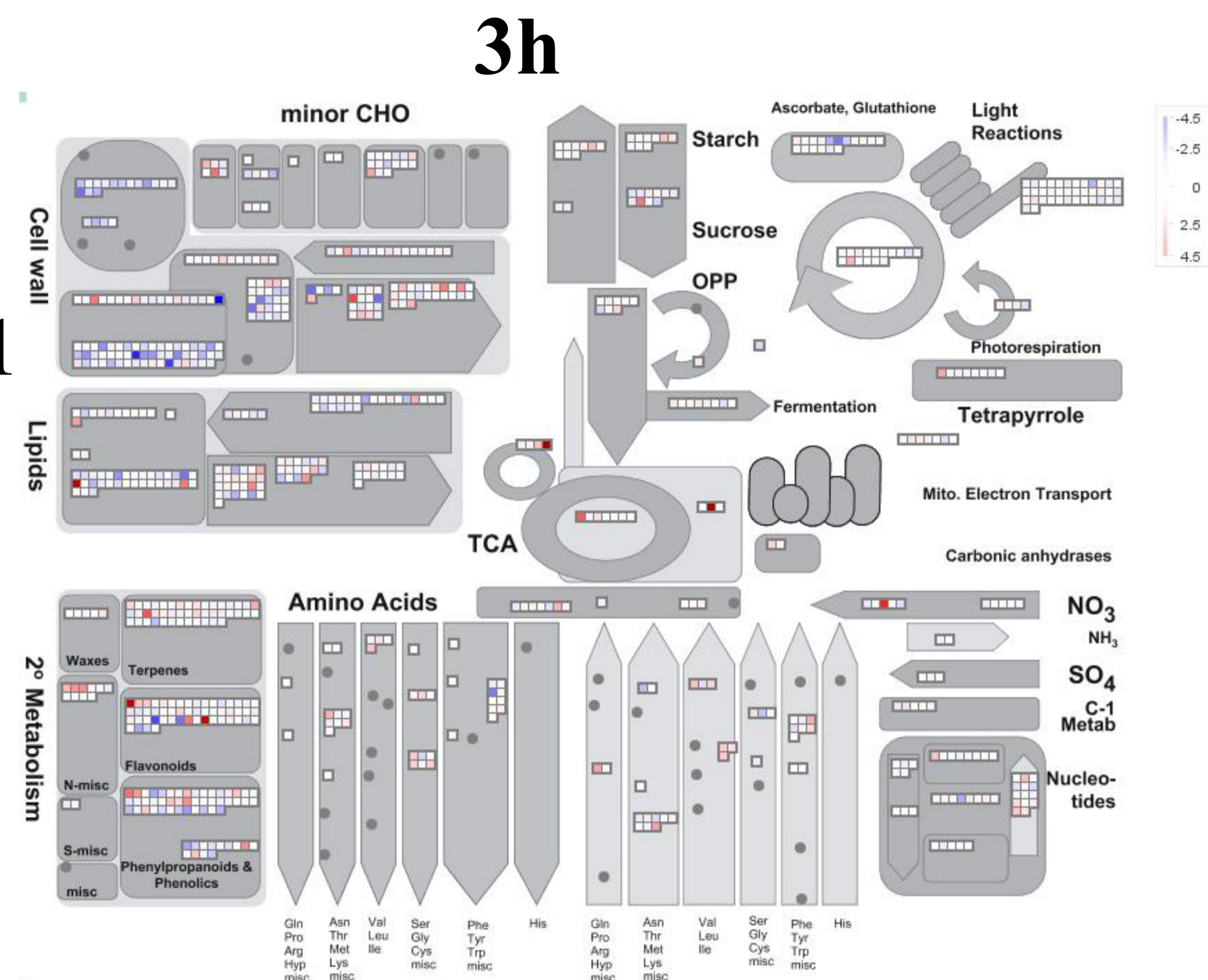

24h

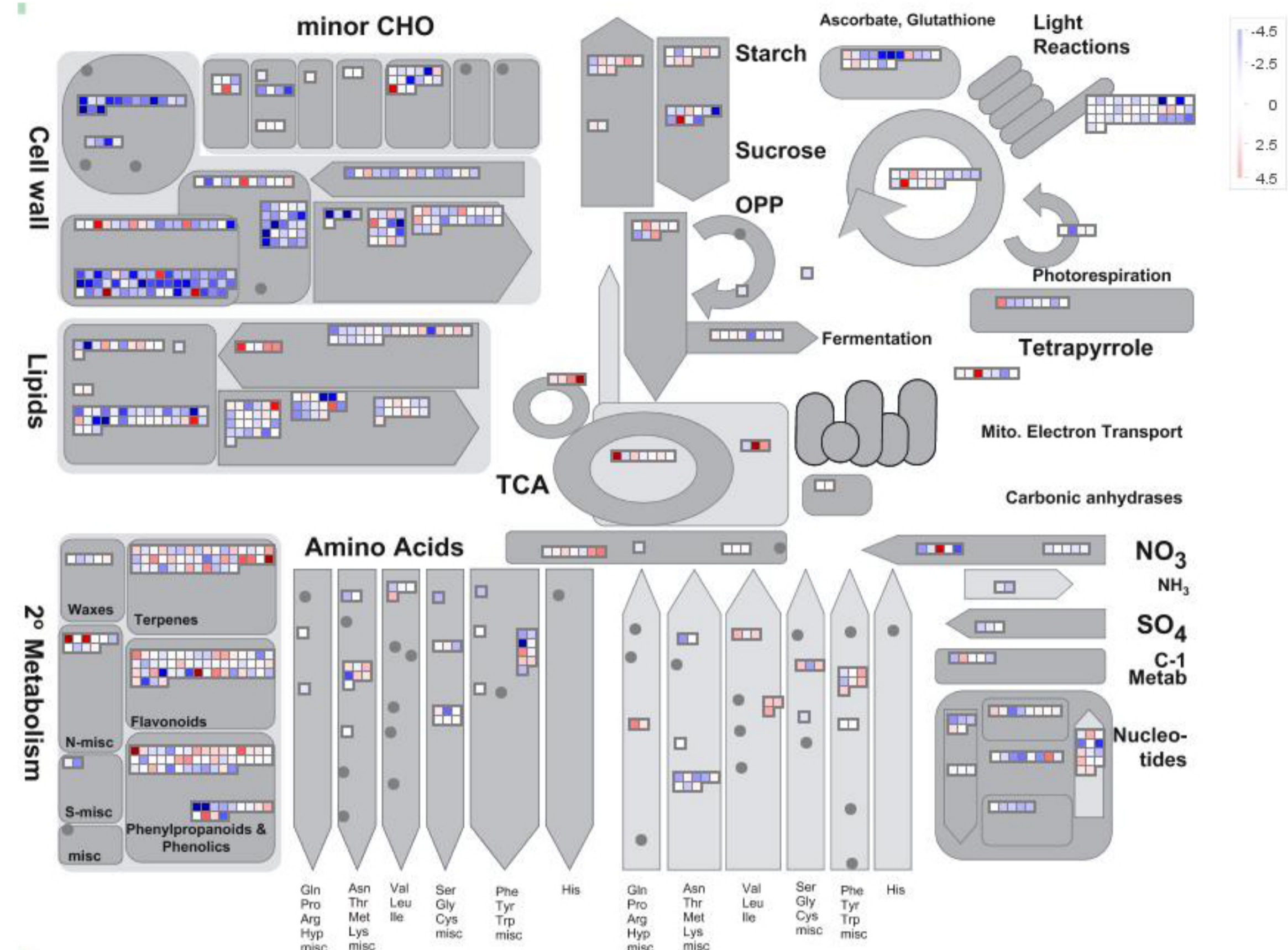

Re24h

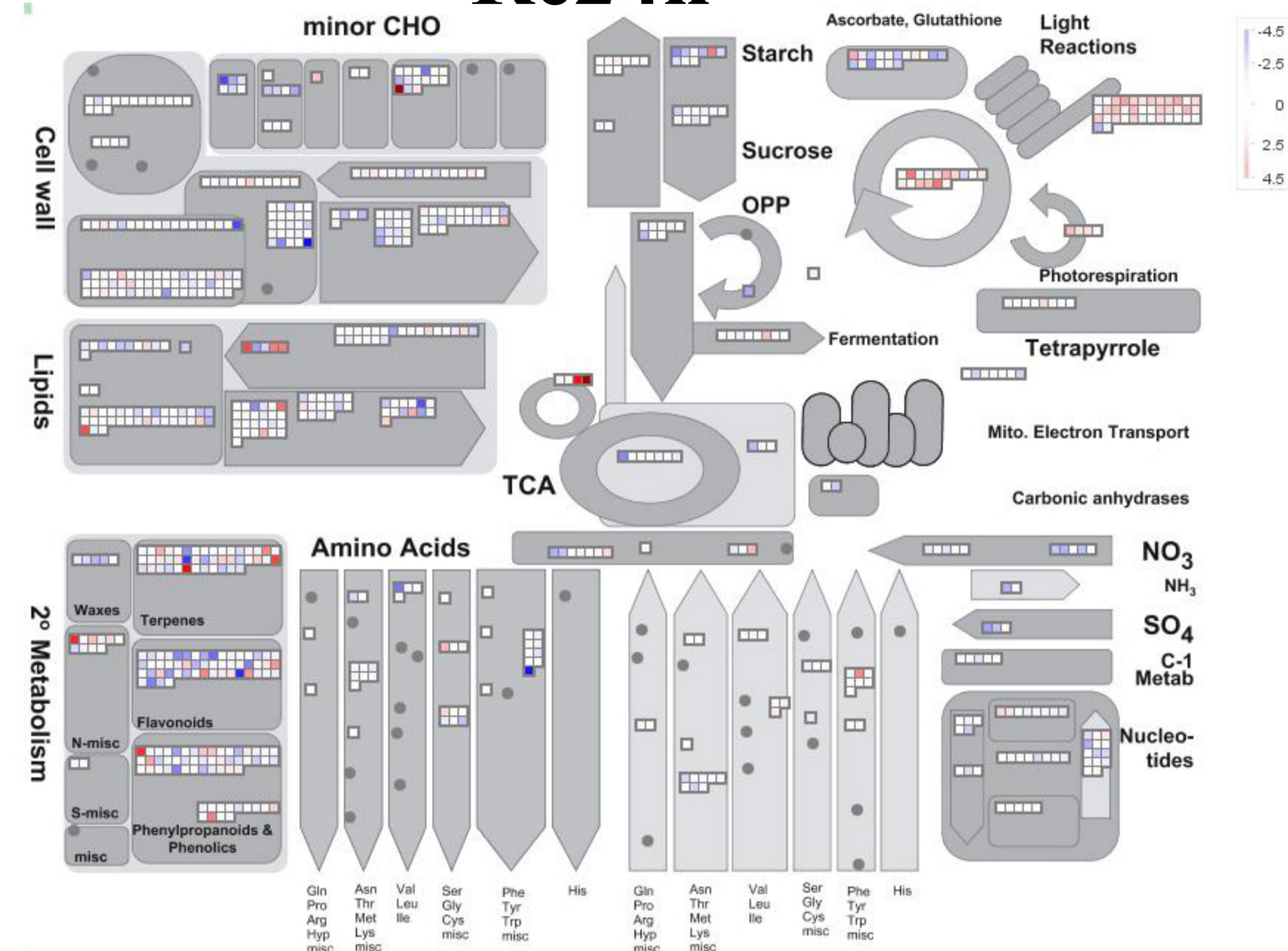

TNG67

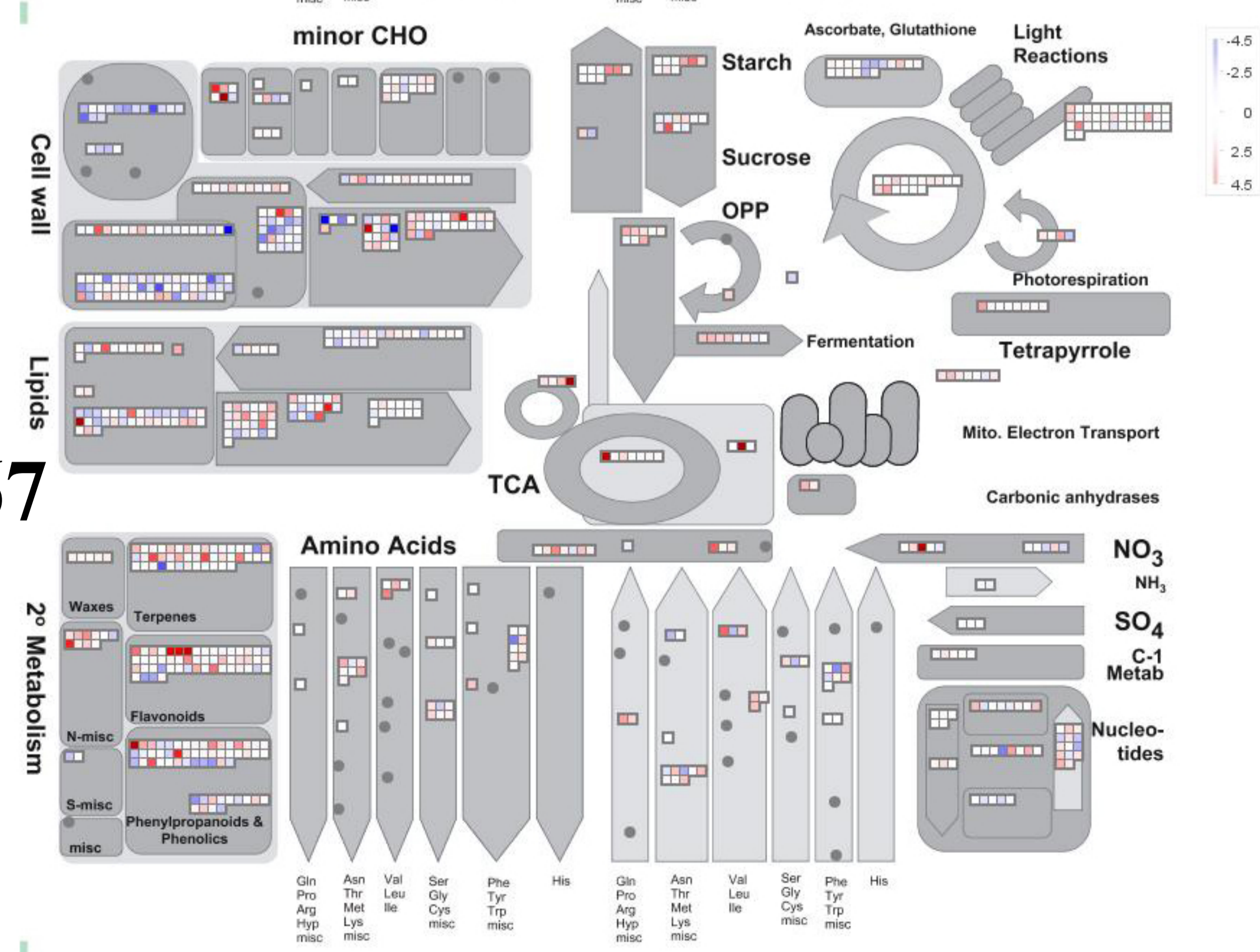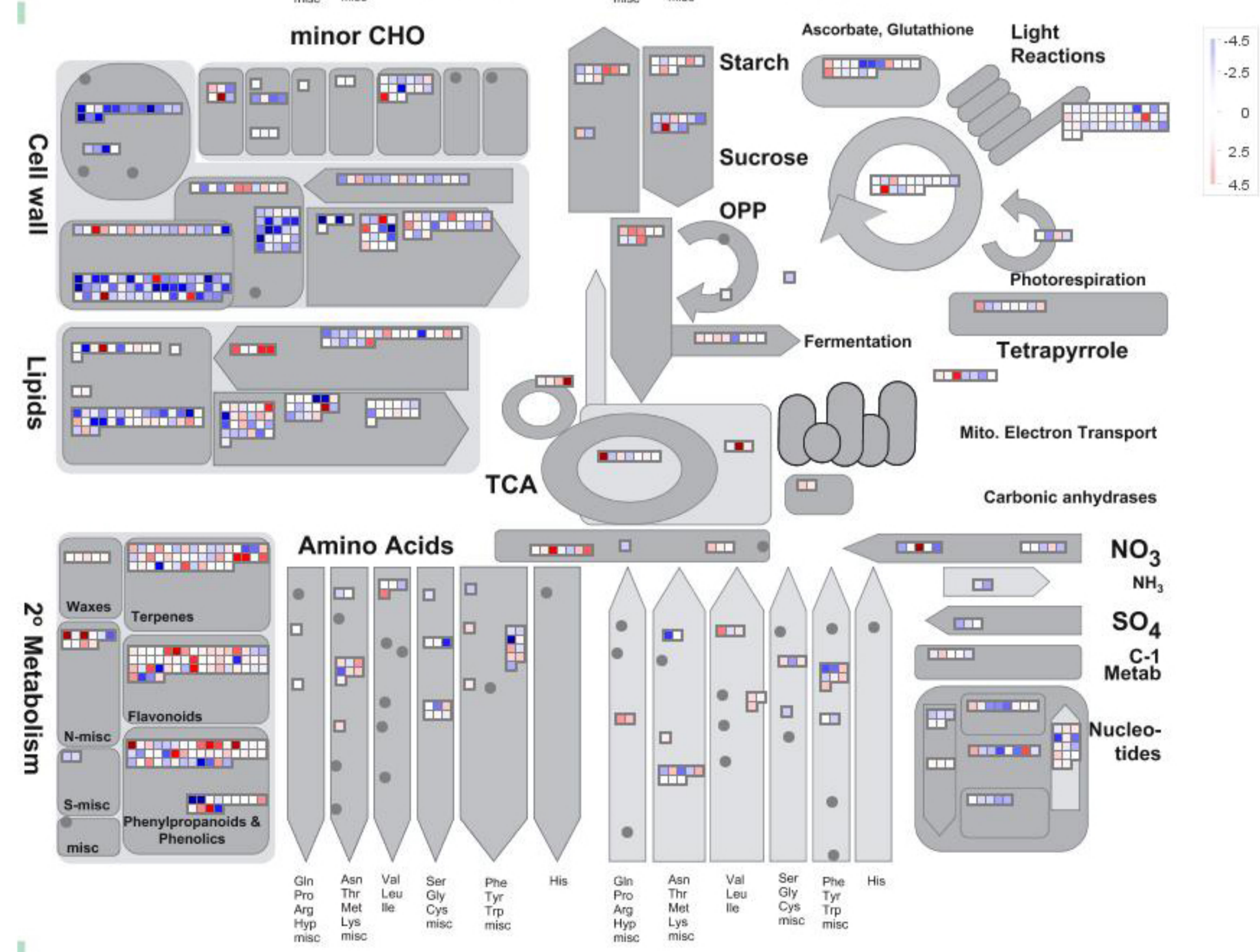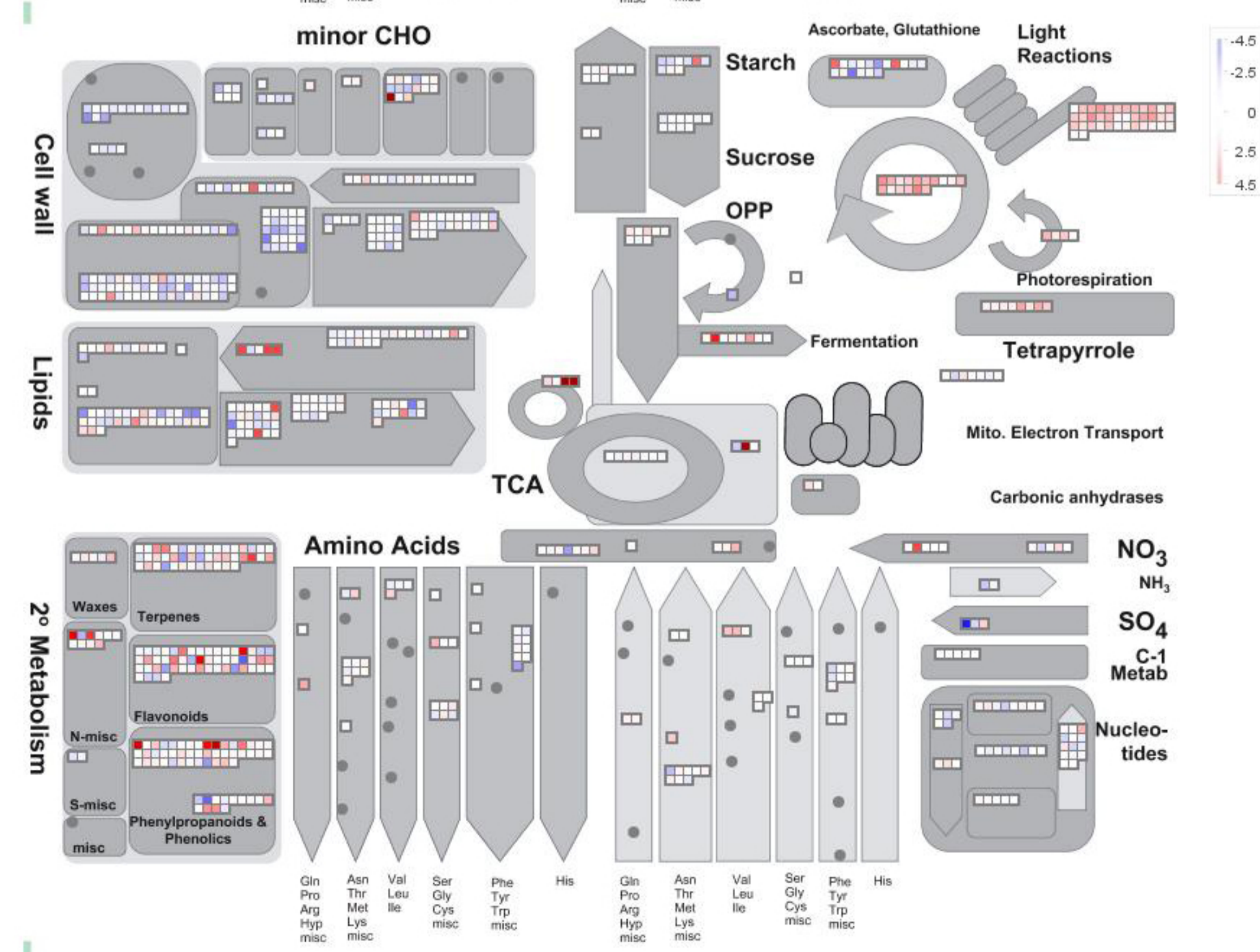

Supplement: Supplementary file 1 [file plants-08-00064-s001.zip › sup/Fig S8.pdf]
